# ﻿Revision of the millipede family Dalodesmidae in Madagascar, with descriptions of two new Malagasy species of *Dalodesmus* Cook, 1896 (Diplopoda, Polydesmida)

**DOI:** 10.3897/zookeys.1223.139346

**Published:** 2025-01-08

**Authors:** Thomas Wesener, Nesrine Akkari, Sergei I. Golovatch

**Affiliations:** 1 Zoological Research Museum Alexander Koenig, Leibniz Institute for the Analysis of Biodiversity Change (LIB), Adenauerallee 127, D-53113 Bonn, Germany Leibniz Institute for the Analysis of Biodiversity Change Bonn Germany; 2 Third Zoological Department, Natural History Museum Vienna, Burgring 7, 1010 Vienna, Austria Natural History Museum Vienna Vienna Austria; 3 Institute of Ecology and Evolution, Russian Academy of Sciences, Leninsky prospekt 33, Moscow 119071, Russia Institute of Ecology and Evolution, Russian Academy of Sciences Moscow Russia

**Keywords:** Endemism, key, Madagascar, map, new synonymies, taxonomy

## Abstract

The family Dalodesmidae Cook, 1896 in the fauna of Madagascar is reviewed and shown to presently encompass eight species in three genera: *Dalodesmus* Cook, 1896 (six species), *Eutubercularium* Brölemann, 1916 (one species), and *Phymatodesmus* de Saussure & Zehntner, 1897 (one species). These genera are diagnosed, and their respective species keyed, all being endemic to Madagascar proper and/or the immediately adjacent islets of Nosy Be and/or Nosy Sakatia. *Dalodesmus* currently contains six species, including two new, all supplied either with brief descriptive notes and available iconography or extensive descriptions and new illustrations, as follows: *D.hamatus* (Brandt, 1841), from an unspecified locality in Madagascar, now redescribed from a male specimen from Makira, northeastern Madagascar; *D.odontopezus* (Attems, 1898), from Nosy Be Isle; *D.orator* Hoffman, 1974, from Ambohimitombo, central Madagascar; *D.tectus* Cook, 1896 (= *D.hova* (de Saussure & Zehntner, 1897), **syn. nov.**), originally described from an unspecified locality in central Madagascar, with a male type and additional males identified as *D.hova* recorded from the Andasibe National Park (= Périnet) in east-central Madagascar. Additionally, two new species are described, *D.speophilus***sp. nov.**, from Grotte de Anjohibe, northwestern Madagascar, and *D.kompantsevi***sp. nov.**, from Montagne d’Ambre, northern Madagascar. Both the type species of *Dalodesmus* and its synonym *Tubercularium* Attems, 1898, *D.tectus* and *D.odontopezus*, respectively, are partly revised and illustrated based on holotypes, while *D.hamatus* is duly described and illustrated based on the first discovery of a male. Both *Eutubercularium* and *Phymatodesmus* are still monospecific, comprising only *E.voeltzkowi* (Mesibov, Wesener & Hollier, 2018), from Nosy Be Isle, and *P.sakalava* (de Saussure & Zehntner, 1901), from Andasibe, respectively. The latter species is fully redescribed and illustrated for the first time from male material.

## ﻿Introduction

The basically austral millipede family Dalodesmidae Cook, 1896 currently contains approximately 55 genera and more than 250 described species. The distribution pattern of the family is highly disjunct, ranging across the Southern Hemisphere: New Caledonia, New Zealand, Australia, southern Africa and Madagascar, and southern South America (Enghoff et al. 2015). Mesibov (2017) has since summarised the dalodesmid fauna of southern South America (Chile with the adjacent parts of Argentina and Brazil), recording 12 accepted genera and 52 species.

Madagascar, despite its huge size and outstanding biogeographic importance, is especially species-poor in indigenous members of the largest diplopod order Polydesmida. All species are contained in two endemic genera of Dalodesmidae (Wesener and Enghoff 2022). *Dalodesmus* Cook, 1896, the type genus of the family, is known to currently comprise six species, whereas *Phymatodesmus* de Saussure & Zehntner, 1902 is monospecific (Wesener and Enghoff 2022). Sometimes a third and also monospecific genus of the Dalodesmidae, *Eutubercularium* Brölemann, 1916, has occasionally been added to the list (Hoffman 1980).

The history of taxonomic research on Malagasy dalodesmids is unusually convoluted (e.g., Mesibov et al. 2018). Prompted by the recent discovery of two new species of *Dalodesmus* in northern Madagascar, we present a modern revision of the family and an illustrated identification key based on freshly collected and historical specimens. The present paper includes the record and first description of the male of *D.hamatus*, a partial revision of the holotypes of both *D.tectus* Cook, 1896, the type species of *Dalodesmus*, and *D.odontopezus* (Attems, 1898), and the description and the first illustration of somatic characters in the former two species.

## ﻿Materials and methods

The material underpinning the present contribution is based on the holotypes of *D.tectus* and *Phymatodesmussakalava*, as well as four fresh collections (by Taiti and Bartolozzi from the MZUF, by Telnov from the NHML, by the late Kompantsev, and by Spelzhausen during her ecological study (see Spelzhausen et al. 2020), each containing a new species or the first male specimen needed for a proper redescription.

The images of *D.kompantsevi* sp. nov. were taken with a Canon EOS 5D digital camera and stacked using Zerene Stacker software. Final image processing was performed with Adobe Photoshop CC. The pictures of *D.hova* from Fort Dauphin and the holotype slide containing the gonopods of *D.odontopezus* were taken with a Nikon DS-Ri2 camera mounted on a Nikon SMZ25 stereo microscope, using NIS-Elements Microscope Imaging Software with an Extended Depth of Focus (EDF) patch. The photographs of *Phymatodesmussakalava*, *D.tectus*, *D.hamatus*, and *D.speophilus* sp. nov. were obtained using a Leica Z6 Imaging System with the software AutoMontage.

For scanning electron microscopy (SEM), the samples were dehydrated via ethanol baths with ascending concentrations, mounted on stubs, and dried overnight. The stubs were sputter-coated with 100 nm of gold in a Hummer VI (Anatech, USA) sputtering system and observed under a Zeiss Sigma 300 VP scanning electron microscope.

The distribution map was generated using QGIS 3.38.3 ‘Grenoble’ and processed applying Photoshop CS6.

In the synonymy section, D stands for a description or descriptive notes, K for the appearance in a key, N for nomenclatural issues, R for a new record or new records, and L for merely a listing.

List of museum acronyms:

**MHNG** Muséum d’histoire naturelle de la Ville de Genève, Geneva, Switzerland

**MZUF** Museum «La Specola», Florence, Italy

**NHMW** Naturhistorisches Museum Wien, Austria

**SMF** Senckenberg Museum of Natural History, Frankfurt/M., Germany

**ZFMK** Zoologisches Forschungsinstitut und Museum Alexander Koenig, Germany

**ZISP** Zoological Institute, Russian Academy of Sciences, St.-Petersburg, Russia

**ZMB** Zoological Museum of the Humboldt University in Berlin, Germany

**ZMUM** Zoological Museum, State University of Moscow, Russia

## ﻿Results

### ﻿Taxonomy

**Order Polydesmida Leach, 1815**

**Family Dalodesmidae Cook, 1896**

**Note.** Following Jeekel (1965, 1971), Hoffman (1980), and Wesener and Enghoff (2022), the Malagasy dalodesmid fauna comprises only three genera: *Dalodesmus* Cook, 1896; *Eutubercularium* Brölemann, 1916, and *Phymatodesmus* de Saussure & Zehntner, 1902.

#### ﻿Genus Phymatodesmus

Taxon classificationAnimaliaPolydesmidaVaalogonopodidae

de Saussure & Zehntner, 1902

C224EF3B-C055-580B-891F-B145147F7102

Phymatodesmus de Saussure & Zehntner, 1902; type species Polydesmussakalava de Saussure & Zehntner, 1897, by monotypy. See Mesibov et al. (2018) for the convoluted taxonomic history of this generic name.

##### Diagnosis.

Body pale reddish, subcylindrical, much shorter than those of *Dalodesmus* and *Eutubercularium*, only ca 10 mm long, 1.0–1.05 mm wide. Paraterga very narrow, declivous and subrectangular, unlike in *Dalodesmus* and *Eutubercularium* where the paraterga are conspicuous and greatly expanded. *Phymatodesmus* differs from both *Eutubercularium* and *Dalodesmus* in the presence of circular cones/tuberculations (vs large, oval to polygonal, often irregular, piligerous tuberculations or areations), and in the 2+2 setae on the paraprocts borne on distinct knobs (not being borne on distinct knobs in *Eutubercularium* and *Dalodesmus*).

##### Remark.

Hoffman (1980) tentatively listed this genus in the family Vaalogonopodidae, *vs*Dalodesmidae in recent species lists (Enghoff 2003; Wesener and Enghoff 2022), a placement we can confirm with the first description of the male.

#### ﻿ Phymatodesmus sakalava

Taxon classificationAnimaliaPolydesmidaVaalogonopodidae

(de Saussure & Zehntner, 1897)

51691BCD-5073-5AB5-A5F1-FFC3AC39BCB4

Figs 1 , 2 , 3

Polydesmus sakalava de Saussure & Zehntner, 1897: plate 5, fig. 22 (figure and caption only).
Polydesmus (Phymatodesmus) sakalava – de Saussure and Zehntner 1902: 95 (D).
Eutubercularium sakalava – Brölemann 1916: 605 (D); Hollier and Wesener 2017: 62 (L).
Dalodesmus sakalava – Jeekel 1965: 238 (L); Hoffman 1974: 230 (L); Golovatch and Hoffman 1989: 162 (L).
Phymatodesmus sakalava – Attems 1940: 490 (L); Enghoff 2003: 623 (L); Mesibov et al. 2018: 389 (N); Wesener and Enghoff 2022: 926 (L).
Dalodesmidae sp. – Spelzhausen et al., 2020: 4 (L).

##### Note.

As iterated above, the confusion de Saussure and Zehntner (1897, 1901, 1902) created by the initial usage of the specific epithet *sakalava* for two species of *Polydesmus* Latreille, 1761 has been resolved only recently (Mesibov et al. 2018). While the original type locality of the species is unknown, Franz Sikora is known to have collected mainly around the capital, Antananarivo. The freshly collected male specimen comes from Andasibe (Spelzhausen et al. 2020), one of the largest remaining blocks of natural vegetation, on the old way from the coast to the capital.

##### Material examined.

• ♀ ***holotype***, fragmented (MHNG), ‘Madagascar’, coll. Sikora • 1 ♂, ZFMK MYR 12217; Madagascar, Moramanga District, Andasibe National Park (= Périnet), Analamazaotra Forest Station, secondary forest, *Eucalyptus* 1909 plantation, sifted leaf litter, IV.2017, L. Spelzhausen and G. Rakotonirina leg.

##### Brief description.

**Figure 1.**
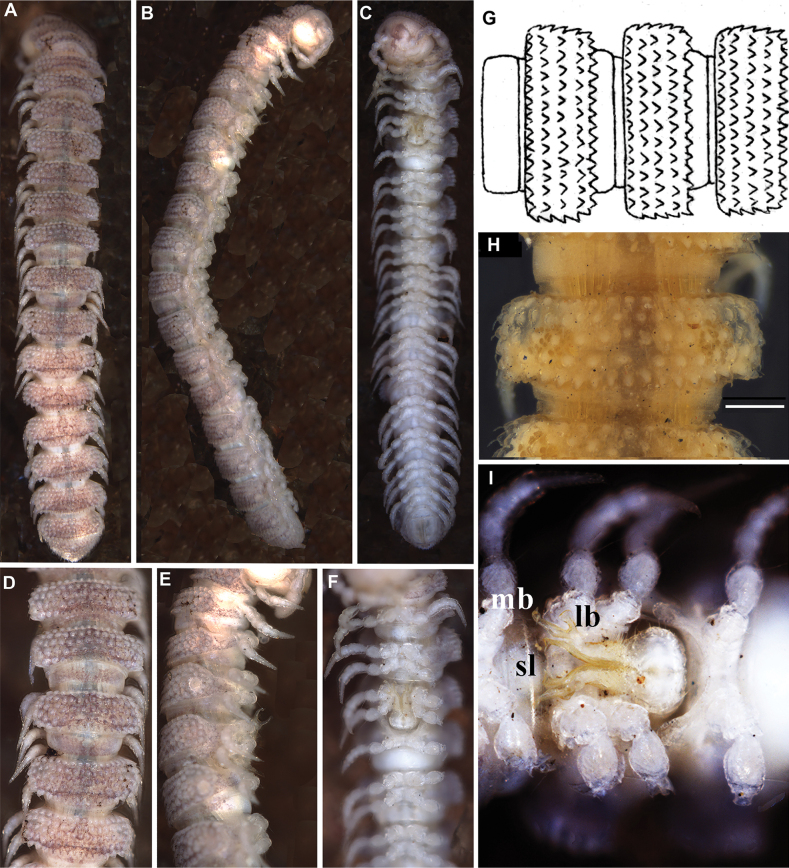
*Phymatodesmussakalava* (de Saussure & Zehntner, 1897), ♀ holotype MHNG (G, H), ♂ specimen (ZFMK MYR12217). Multi-layer photographs and drawings **A** habitus, dorsal view **B** habitus, lateral view **C** habitus, ventral view **D** detail of midbody rings, dorsal view **E** detail of anterior body rings and gonopod, lateral view **F** detail of anterior rings and gonopods, ventral view **G** drawing from the original description in de Saussure and Zehntner (1897)**H** detail of body ring, ♀ holotype **I** gonopod still attached, ventral view. Abbreviations: lb = lateral branch; mb = mesal branch; sl = solenomere branch. Scale bar: 0.3 mm (**H**).

(After de Saussure and Zehntner 1897, 1902.) A single line drawing (Fig. 1G) and its caption reading “*Polydesmussakalava*” in de Saussure and Zehntner (1897) serve as the original description. Combined with a later verbal description of the adult ♀ holotype, by de Saussure and Zehntner (1902), the following relevant information can be obtained: body pale reddish, subcylindrical, 10 mm long, 1 mm wide, with a strongly convex dorsum and very narrow, declivous, and subrectangular paraterga.

*Phymatodesmus* s*akalava* can be readily distinguished from other known Malagasy Dalodesmidae: the small size, a subcylindrical body, the mostly small, conical, and sharp tuberculations on midbody metaterga arranged in four unusually regular transverse rows, and strikingly small, narrow, and rectangular paraterga.

##### First description of the male.

Length ca 10 mm, width of midbody pro- and metazona 0.75 and 1.05 mm, respectively.

Colouration (freshly preserved in ethanol) brown; prozona, basal parts of legs, mandibles, and paraprocts paler. Epicranium grey, antennae and apical parts of legs faded grey.

Body with 20 rings. Tegument mainly dull (Fig. 1A–F), microgranulate to microtuberculate throughout (Fig. 1G, H), even surfaces of prozona and of metazona below paraterga finely microgranulate, sterna granulate.

Head also densely microtuberculate or granulate throughout, micropilose; epicranial suture thin, but distinct; genae squarish, set off ventrally from gnathochilarial stipes by a small, but evident ridge. Interantennal isthmus ~ 2 × diameter of antennal socket (Fig. 1C).

Antennae very short and rather clavate, in situ reaching back past ring 2 when stretched dorsally, very densely setose and microgranulate. In length, antennomere 6 > 5 = 3 > 4 > 2 > 1 = 7; antennomere 6 the largest and the highest, antennomeres 5 and 6 each with a small, round, distodorsal knob, most likely beset with sensory cones.

**Figure 2.**
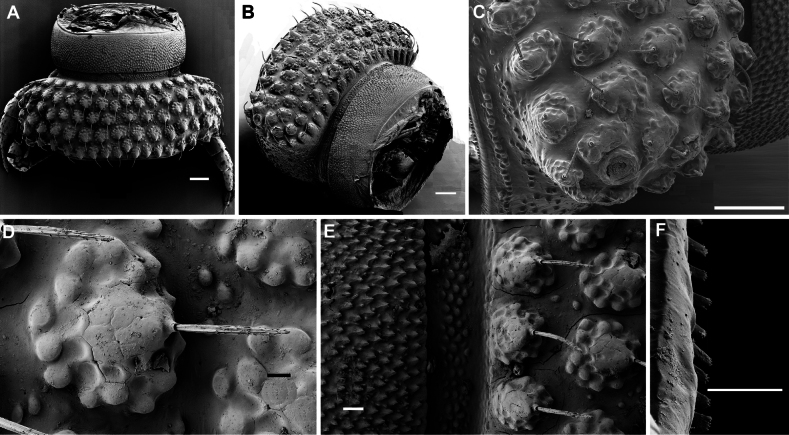
*Phymatodesmussakalava* (de Saussure & Zehntner, 1897), ♂ specimen (ZFMK MYR12217). SEM micrographs **A** midbody ring with pro- and metazonite, dorsal view **B** midbody ring, lateral view **C** midbody ring, paranota with ozopore **D** details of surface structures, dorsal view **E** detail of surface structures of prozonite and metazonite **F** endotergum. Scale bars: 100 µm (**A–C**); 10 µm (**D**); 20 µm (**E**); 10 µm (**F**).

In width, collum = head < ring 2 > 3 = 4–16; thereafter body gradually tapering towards telson (Fig. 1A–C). Collum transversely suboval, regularly and broadly rounded laterally, densely tuberculate, most tuberculations being circular, evident, equipped with very short, mostly subclavate setae and arranged in 15–17 lateral, 8–9 transverse, rather irregular, arcuated rows. Metaterga 2–4 narrow, each with four, similar, transverse, arcuated, circular rows of setigerous tubercles, following metaterga each largely with five such rows (Fig. 2A, B). Paraterga short, rectangular, strongly declivous, posterior margin straight (Fig. 2B, C). Lateral margin of paraterga beset with 5–6 similarly circular, setigerous (Fig. 2D) tubercles/lobulations. Ozopores inconspicuous, opening laterally near penultimate lateral lobulation on pore-bearing rings 5, 7, 9, 10, 12, 13, 15–19. Strictures between pro- and metazona narrow and rather deep, nearly smooth (Fig. 2E).

Telson: Epiproct small, conical, and subtruncate at tip. Hypoproct trapeziform, with 1+1 setae borne on distinct oblong knobs at caudal margin. Paraprocts with 2+2 setae on triangular and projecting knobs (Fig. 2C).

Limbus very thin, small, and entire. Neither an axial line nor pleurosternal carinae (Fig. 1A–C). Posterior margin of metazona a row of dense, elongate, apically microdenticulate (with 6–9 indentations (Fig. 2F), subrectangular projections (Fig. 2F)).

Gonopodal aperture roundly pentagonal, large, taking up ~ 2/3 width of metazonum 7, clearly open and drawn into metazonum 6 (Fig. 1I).

First two leg pairs shorter and thicker than other legs. Midbody legs incrassate, medium in length, as long as body height, with small, stout, abundant, and usually curved setae with admixture of sphaerotrichomes ventrally on all podomeres; gonopores on coxae 2 inconspicuous, prefemora not bulged laterally; claws simple, very small; in length, tarsus > femur > prefemur > coxa > tibia = postfemur.

**Figure 3.**
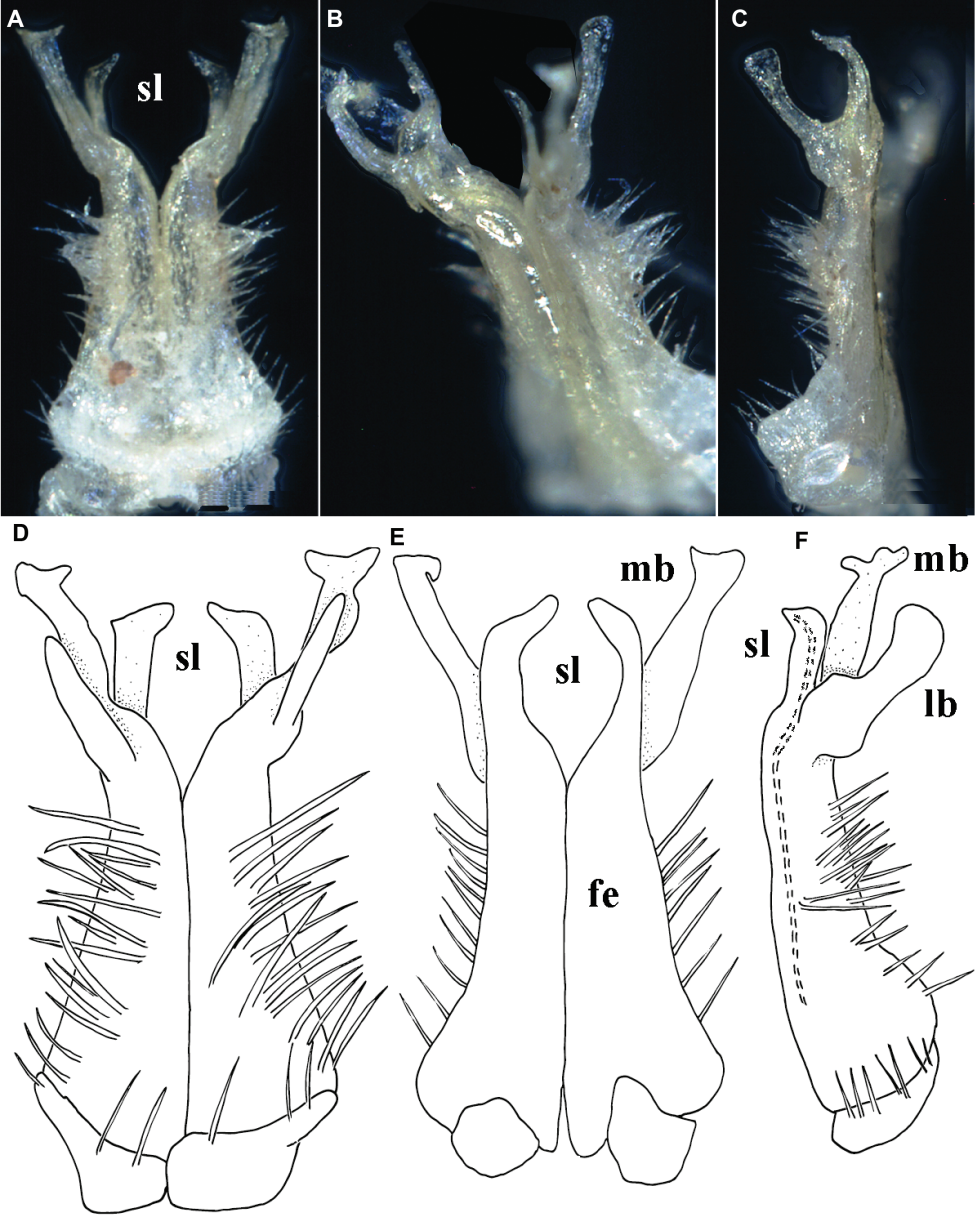
*Phymatodesmussakalava* (de Saussure & Zehntner, 1897), ♂ specimen (ZFMK MYR12217). Multi-layered photographs and drawings, gonopod **A** posterior view **B** lateral and mesal view **C** lateral view **D** posterior view **E** anterior view **F** lateral view. Abbreviations: fe = femorite; lb = lateral branch; mb = mesal branch; sl = solenomere branch.

Gonopods (Figs 1I, 3A–F) relatively simple. Both coxite and prefemorite very short, fused medially, prefemorite setose. Femorites (fe) contiguous medially, densely setose both ventrally and laterally, rather stout (~ 2 × as long as acropodites), suberect and clearly flattened dorsoventrally. Acropodite tripartite, divided into a dorsomedial, long, simple and subsecuriform solenomere (sl), a simple and subspiniform lateral branch (lb), both sl and lb being subequal in length, but clearly shorter than the longest, simple, rather finger-shaped, apically roundly and irregularly trifid, mesal branch (mb).

##### Remarks.

Aside from the very obvious differences between *Phymatodesmus* and *Dalodesmus* in the development and shape of paraterga, clear-cut discrepancies also concern leg lengths (in *Phymatodesmus*, the whole legs, especially both postfemora and tibiae are shorter), the antennae are also shorter, the collum is as wide as the head, the head is completely micropilose, including the epicranium (vs glabrous in *Dalodesmus*), the paraprocts of the telson lack triangular setiferous knobs/projections (vs 2+2 setae borne on knobs on the paraprocts), and the 1+1 setae on the hypoproct are borne on prominent knobs and better separated (vs placed closer to one another in *Dalodesmus*).

#### ﻿Genus Eutubercularium

Taxon classificationAnimaliaPolydesmidaDalodesmidae

Brölemann, 1916

F6094B2C-A9F8-500A-8B3E-4572849D583D

Eutubercularium Brölemann, 1916; type species Pterodesmussakalava de Saussure & Zehntner, 1901 (currently Dalodesmusvoeltzkowi Mesibov, Wesener & Hollier, 2018), by original designation, synonymised with Dalodesmus by Jeekel (1965), resurrected from synonymy following Hoffman (1980).

##### Diagnosis.

26 mm long, 4.5 mm wide, habitus identical to that of *Dalodesmus*, with five rows of transverse, often rather irregular rows of piligerous tuberculations or obliterated areations between always strongly developed and laterally similarly piligerous and crenulate/tuberculate paraterga.

The basic differences from *Dalodesmus* lie only in gonopodal conformation: femorites densely setose all over ventral and lateral sides, relatively stout, only ~ 2 × as long as acropodites and, much like in *Phymatodesmus*, clearly flattened dorsoventrally (vs less strongly setose to nearly bare, much longer, slender and subcylindrical); solenomere (sl) lateral, long, flagelliform, and non-sigmoid (vs medial, short, usually simple and rather rod- or lobe-shaped, often sigmoid), lateral branch (lb) remarkably tripartite and complex (vs lb unipartite and usually simple to rather simple).

##### Remarks.

Brölemann (1916) described *Eutubercularium* based on gonopod differences derived only from the drawings of the species published by de Saussure and Zehntner (1901: figs 8–10) for *Polydesmussakalava* (now *Dalodesmusvoeltzkowi* Mesibov, Wesener & Hollier, 2018). Apparently, the type specimens can no longer be located. Jeekel (1965) hesitantly decided to synonymise *Eutubercularium* with *Dalodesmus*, as the observed differences could be a simple drawing error. Hoffman (1980) listed *Eutubercularium* again as a valid genus, but without explanation, a move not followed in recent species lists (Enghoff 2003; Wesener and Enghoff 2022). Here, we resurrect *Eutubercularium* from synonymy with *Dalodesmus* following Hoffman (1980).

#### ﻿ Eutubercularium voeltzkowi

Taxon classificationAnimaliaPolydesmidaDalodesmidae

(Mesibov, Wesener & Hollier, 2018) comb. nov.

34582356-927F-5F20-B834-4203D77242BF

Fig. 4

Polydesmus (Pterodesmus) sakalava – de Saussure & Zehntner, 1901: 437, figs 8–10 (D).
Polydesmus (Tubercularium) sakalava – de Saussure and Zehntner 1902: 93 (D).
Eutubercularium sakalava – Brölemann 1916: 605 (D); nec Hollier and Wesener 2017: 62 (L, N).
Non Tuberculariumsakalava – Attems 1940: 435, fig. 619 (D, K).
Nec Dalodesmussakalava – Hoffman 1974: 230 (D); nec Golovatch and Hoffman 1989: 162 (L); nec Enghoff 2003: 623 (L).
Dalodesmus voeltzkowi Mesibov, Wesener & Hollier, 2018: 389 (N), nom. nov.
Dalodesmus voeltzkowi – Wesener and Enghoff 2022: 926 (L).

##### Note.

The new name *voeltzkowi* was proposed to dispose of the homonymy within *Polydesmus* Latreille, 1761, that had been created by de Saussure and Zehntner (1902), i.e., to correctly conserve *P.sakalava* de Saussure & Zehntner, 1897 as the older, and therefore valid, name (see below), and to replace *P.sakalava* de Saussure & Zehntner, 1901 as a later and invalid name (Mesibov et al. 2018). No material of *Polydesmussakalava* could be traced at the SMF (P. Jäger, pers. comm. April 2024) and the type locality is Nosy Be (de Saussure and Zehntner 1901)

##### Brief description.

**Figure 4.**
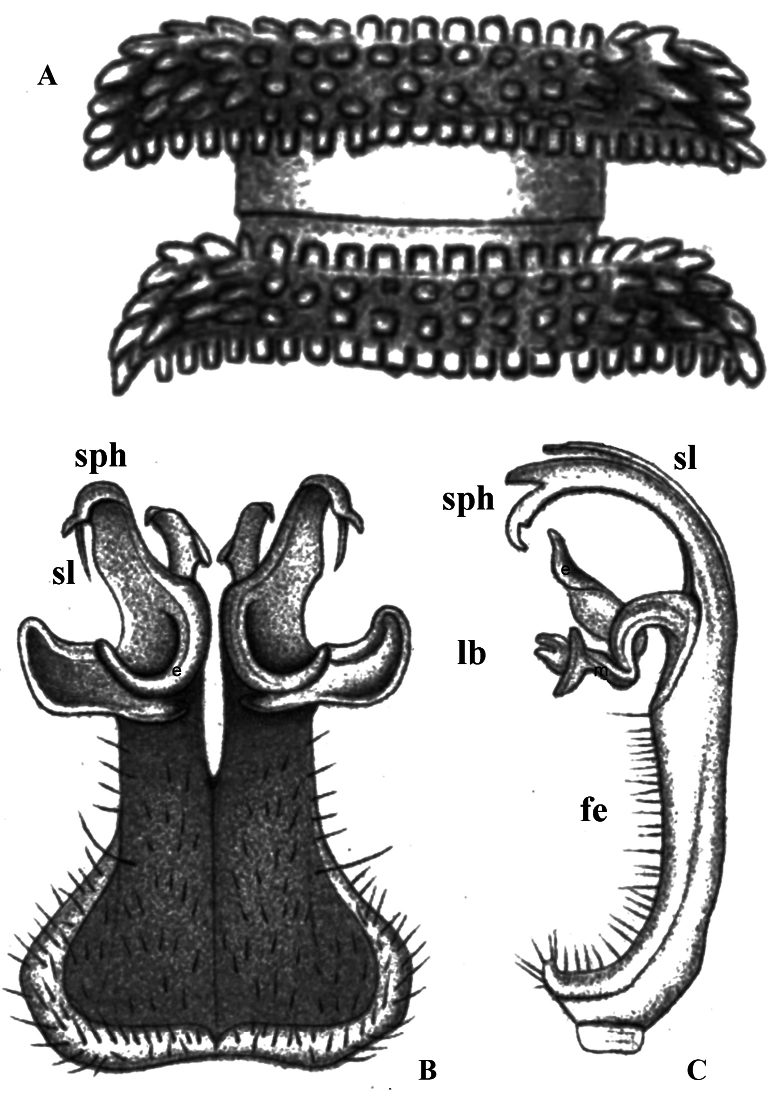
*Eutuberculariumvoeltzkowi* (Mesibov, Wesener & Hollier, 2018), ♂ or ♀. Redrawn original drawings from de Saussure and Zehntner (1901)**A** midbody tergites, dorsal view **B** gonopod, posterior view **C** gonopod, lateral view of acropodite. Abbreviations: fe = femorite; lb = lateral branch; sl = solenomere branch; sph = highest solenophore branch.

(After de Saussure and Zehntner 1901, 1902.) First described in a short text with three drawings (de Saussure and Zehntner 1901: 8–10). Combined with a later verbal description of the adult ♂ holotype, by de Saussure and Zehntner (1902), the following relevant information can be derived: colouration brick red, metaterga castaneous brown, ends of paraterga brick red. Length 26 mm, width 4.5 mm. Paraterga strong, clearly rounded and crenulate laterally, acute caudolaterally. Metaterga with five, transverse rows of conical tuberculations (Fig. 4A).

Gonopods highly elaborate and complex, with coxites and densely setose prefemorites short and fused medially; femorites strongly flattened dorsoventrally, contiguous medially and fused in about basal half, without a sternal rudiment visible ventrally, each femorite setose until acropodite, this latter highly complex, basically 3-branched: (1) what seems to be a long and flagelliform solenomere branch (sl) lying lateral to and supported by (2) the highest solenophore branch (sph), unequally bifid apically, sl and sph both slightly and regularly curved ventrad; and (3), more basally, at apical ~ 1/3, a very complex lateral branch (lb) consisting of an ear-shaped lateral outgrowth (e) with a shelf ventrobasally, a strong, subacuminate, distomesal uncus, regularly curved mesad and lying just distal to (e) shelf, and a twisted mesal lobe (m), directed distally, originating near lb base and with its tip curved ventrad (Fig. 4B, C).

##### Remarks.

It is only the unusually complex gonopodal conformation of *E.voeltzkowi* that allows us to clearly distinguish the genus *Eutubercularium* from the superficially very similar *Dalodesmus*: femorites densely setose all over the ventral and lateral sides, relatively stout, only ~ 2 × as long as acropodites and, much like *Phymatodesmus*, clearly flattened dorsoventrally (vs much longer, slender, and subcylindrical, less strongly setose to nearly bare); solenomere (sl) lateral, long, flagelliform and non-sigmoid (*vs* medial, short, usually simple and rather rod- or lobe-shaped, often sigmoid), lateral branch (lb) remarkably tripartite and complex (vs lb unipartite and usually simple).

#### ﻿Genus Dalodesmus

Taxon classificationAnimaliaPolydesmidaDalodesmidae

Cook, 1896

F2E9C669-2D45-5CED-8CBE-ECC193233FE4

Dalodesmus Cook, 1896; type species D.tectus Cook, 1896, by monotypy.
Tubercularium Attems, 1898; type species: T.odontopezum Attems, 1898, by monotypy, synonymised by Jeekel (1965).
Pterodesmus de Saussure & Zehntner, 1901; invalidly proposed without a type species, also preoccupied by Pterodesmus Cook, 1896; synonymised by Jeekel (1965).

##### Note.

Species included: 6 (including two new described below).

##### Diagnosis.

Body medium-sized, 17–28 mm long and 3.7–5.3 mm wide. Midbody metaterga with 4–6 transverse, often rather irregular rows of piligerous tuberculations or obliterated areations between always strongly developed and laterally similarly piligerous crenulate/tuberculate paraterga, these being sub-horizontal to upturned. Gonopods less elaborate and more simple than in the other genera, both coxites and densely setose prefemorites equally short and fused medially; femorites (fe) mostly slender, subcylindrical, at most only insignificantly flattened sagittally, contiguous medially and fused in basal 1/3, with a sternal rudiment visible ventrally at the very base, bare to sometimes sparsely setose until acropodite, the latter (= acropodite) basically tripartite, a mostly distinct, occasionally branching and only rarely missing solenomere branch (sl) lying between and typically flanked by two or three branches of a rather simple to elaborate solenophore: one medial (mb), this only rarely subdivided into an apical (ab) and a subapical branch (sb), and the other lateral (lb).

*Dalodesmus* differs from *Phymatodesmus* in the much larger size (> 20 mm, vs ~ 10 mm), the strongly developed, apically pointed paratergites (*vs* short and rectangular in *Phymatodesmus*), the presence of large, oval to polygonal, often irregular, piligerous tuberculations or areations (vs circular cones/tuberculations in *Phymatodesmus*), and the 2+2 setae on the paraprocts not being borne on distinct knobs (vs borne on distinct knobs in *Phymatodesmus*).

In gonopodal structure, the genus *Dalodesmus* differs from both *Phymatodesmus* and *Eutubercularium* in the femorites being long and slender, > 2 × as long as acropodites, subcylindrical, diverging in distal 2/3 to 1/2, bare to only poorly setose ventrally and/or laterally (vs femorites stout, only ~ 2 × as long as acropodites, clearly flattened dorsoventrally, contiguous all along or nearly so, and densely setose on both ventral and lateral sides).

#### ﻿ Dalodesmus hamatus

Taxon classificationAnimaliaPolydesmidaDalodesmidae

(Brandt, 1841)

6198C4DD-7512-588A-915C-56609DFC8C73

Figs 5 , 6 , 7 , 8

Polydesmus hamatus Brandt, 1841a: 10–11 (D); Brandt 1841b: 140 (D); Gervais 1847: 114 (D); Attems 1940: 493 (L); Golovatch and Hoffman 2000: 237 (L).
Non Polydesmus (Tubercularium) sakalava – de Saussure and Zehntner 1902: 93 (N).
Dalodesmus hamatus – Golovatch and Hoffman 1989: 160, figs 1–6 (D); 2000: 237 (L); Enghoff 2003: 623 (L); Wesener and Enghoff 2022: 926 (L).

##### Note.

The ♀ holotype, currently housed in the ZISP collection and coming from an unspecified locality, presumably in Madagascar, has been revised, properly redescribed, and illustrated (Golovatch and Hoffman 1989).

##### New material examined.

• ♂ (ZFMK MYR13631), Madagascar, Toamasina Province, Analanjirofo, Makira Natural Park, ca 44.5 km NW of Maroantsetra, Antainambalana River tributary, 1 km around coordinates, 15°4'15"S, 49°34'48"E, 240–670 m, primary lowland rainforest on basalt, 30.VIII.–08.IX.2023, D. Telnov leg. • 2 ♀ (ZFMK MYR13629), same data as previous • 2 ♀ (ZFMK MYR13630), with eggs, same data as male • 1 ♀ (NHML), same data as male.

##### Diagnosis.

Tips of paraterga mostly sharp and projecting past posterior tergal margin, as in *D.odontopezus*, *D.orator*, vs wider and not projecting past rear tergal margin in *D.speophilus* sp. nov., *D.tectus*, and *D.kompantsevi* sp. nov. Colour uniformly dark grey to blackish, paraterga not yellow as opposed to *D.odontopezus* and *D.orator*. Both latter species with a male body length of 26–28 mm that is larger than *D.hamatus* with 20 mm male and 22–24 mm females. See also the key below.

##### Identity of the new material of *D.hamatus*.

**Figure 5.**
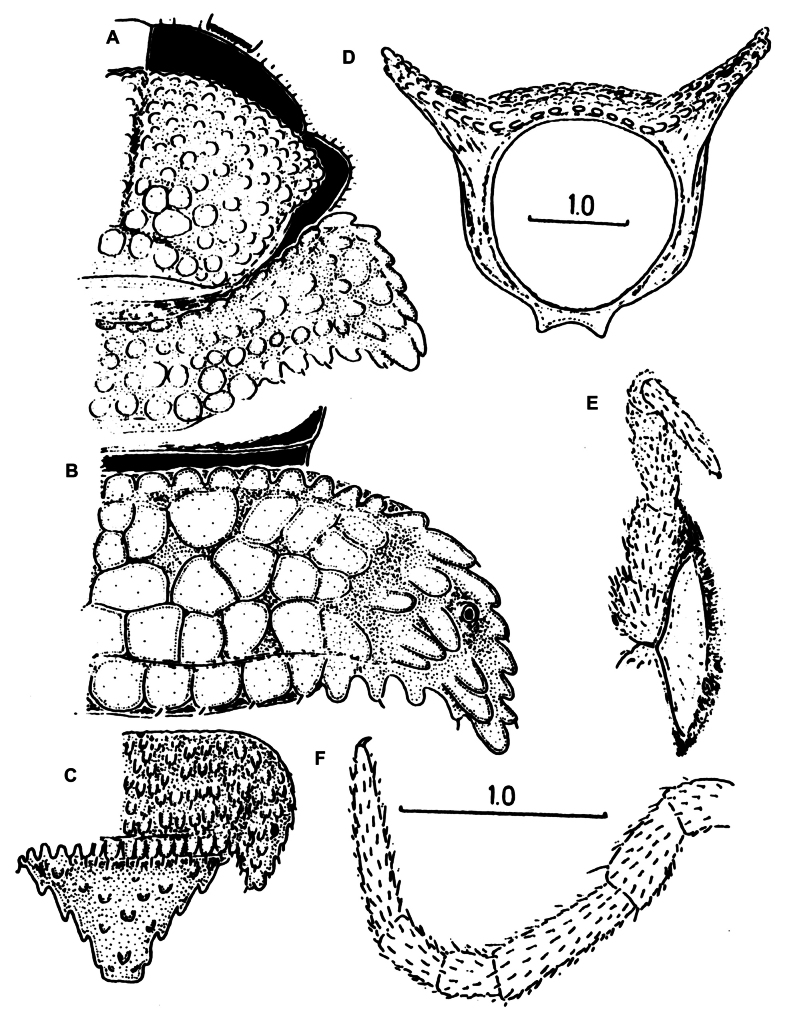
*Dalodesmushamatus* (Brandt, 1841), ♀ holotype **A** anterior part of body, dorsal view **B** right half of metazonum 10, dorsal view **C** posterior end of body, dorsal view **D** body ring 9, oral view **E** leg 2 and epigynal lobe, ventral view **F** midbody leg, lateral view. Scale bars: 1000 µm (after Golovatch and Hoffman 1989).

The ♀ holotype (after Golovatch and Hoffman 1989) has a similar size (length ~ 22 mm, width 2.9 mm) to the newly discovered material. Its colouration is rusty brown (dry specimen, probably faded). The habitus with the paraterga clearly upturned above the dorsum (Fig. 5A–D), dorsal surface between midbody paraterga largely areate, polygonal bosses mostly being clearly obliterated (Fig. 5B), this being identical to the new material. While the type locality of *D.hamatus* is just “Madagascar”, the newly discovered locality in Makira Natural Park also included specimens of the very large (> 200 mm) spirostreptid *Analacostreptussculptus* (de Saussure & Zehntner, 1902), another species otherwise known only from “Madagascar”.

##### Redescription.

(Based on fresh material from Makira.) Length ~ 20 mm (*n* = 1), width of midbody pro- and metazona 1.7 and 3.9 mm (*n* = 1), respectively (♂), ♀♀ 22–24 mm long (*n* = 3), width of prozona 1.9–2.3 mm (*n* = 1), of metazona 4.1–4.3 mm (*n* = 4).

**Figure 6.**
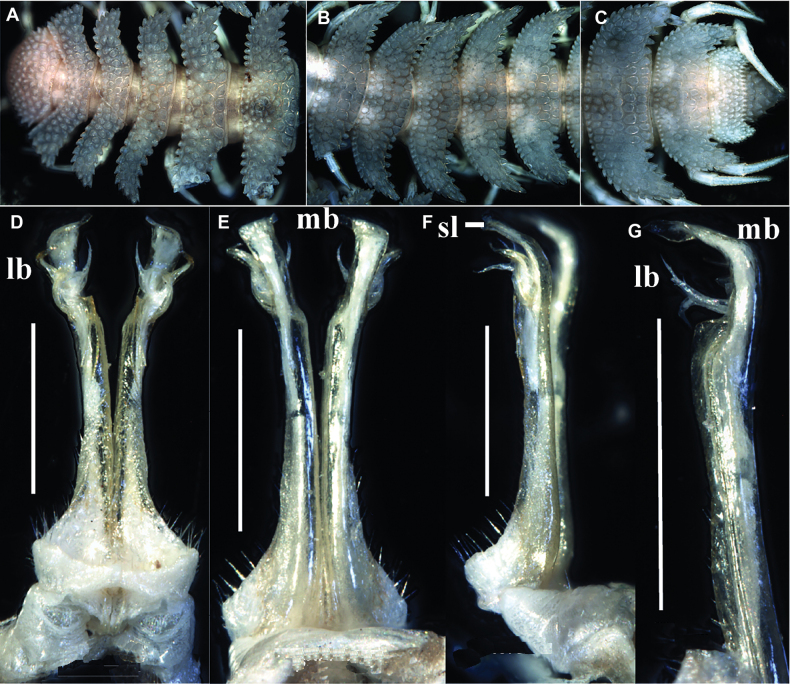
*Dalodesmushamatus* (Brandt, 1841), ♂ from Makira (ZFMK MYR13631). Multi-layer photographs **A** anterior body, dorsal view **B** midbody, dorsal view **C** telson, dorsal view **E** gonopods, posterior view **E** gonopods, anterior view **F** gonopods, lateral view **G** right gonopod, mesal view. Abbreviations: lb = lateral branch; mb = mesal branch; sl = solenomere branch. Scale bars: 1000 µm.

Colouration, after less than 6 months of preservation in alcohol, dark grey to blackish, collum faded brown, head brown, epicranium grey, legs pale grey; antennae dark brown (Fig. 6A–C).

**Figure 7.**
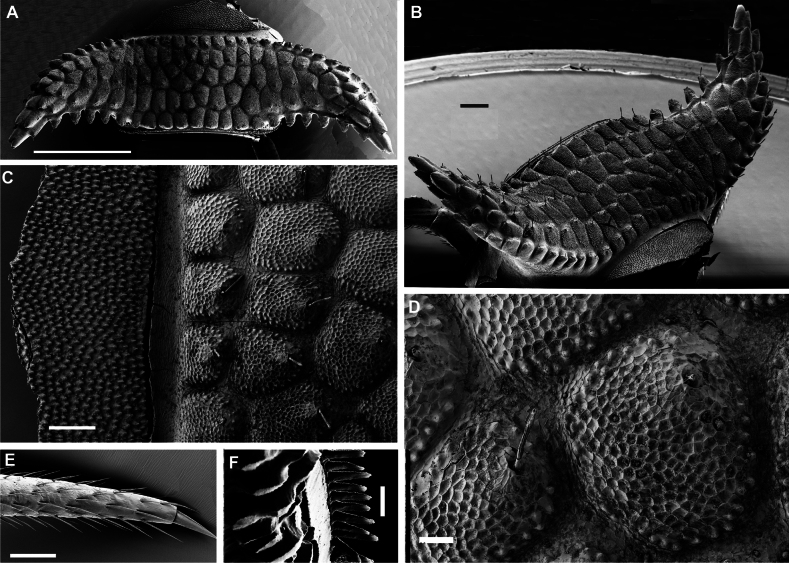
*Dalodesmushamatus* (Brandt, 1841), ♂ from Makira (ZFMK MYR13631). SEM micrographs **A** midbody ring with pro- and metazonite, dorsal view **B** midbody ring, lateral view **C** details of surface structures of prozonite and metazonite **D** details of surface structures, dorsal view **E** tarsus of midbody leg **F** endotergum. Scale bars: 1000 µm (**A**); 200 µm (**B**); 100 µm (**C, E**); 30 µm (**D**); 0 µm (**F**).

Body with 20 rings. Tegument mainly dull, microgranulate to microtuberculate throughout (Figs 5A–D, 6A–C, 7A–D), even surfaces of prozona and of metazona below paraterga finely microgranulate, sterna granulate. Head also densely microtuberculate or granulate throughout, micropilose up to level of antennae; epicranial suture thin, but distinct; genae squarish, set off ventrally from gnathochilarial stipes by a small, but evident ridge. Interantennal isthmus ~ 2 × diameter of antennal socket. Antennae short and rather clavate, in situ reaching in both sexes back past ring 4 when stretched dorsally, very densely setose and microgranulate. In length, antennomere 6 > 3 > 5 > 4 = 2 > 1 = 7; antennomere 6 the largest and the highest, antennomeres 5 and 6 each with a small, round, distodorsal knob, most likely beset with sensory cones. In width, collum ≤ head < ring 2 =3 < 4–16; thereafter body gradually tapering towards telson (Fig. 6A–C). Collum transversely suboval, regularly and broadly rounded laterally, densely tuberculate, most tubercles slightly oblong-oval, evident, equipped with very short, mostly subclavate setae and arranged in 16–17 lateral, 6 transverse, rather irregular, arcuated rows. Metaterga 2–4 narrow, each with 3–4 similar transverse arcuated rows of setigerous tubercles, following metaterga each largely with 4–5 such rows (Fig. 7A, B). Paraterga well-developed, set high (mostly at upper ¼ body), upturned to subhorizontal, thus leaving the dorsum only faintly convex (Fig. 7B); anterior and posterior margins of paraterga 2 and 3 clearly drawn forward and caudad, respectively, following paraterga drawn increasingly only caudad (Fig. 6A–C); caudal corners sharp, produced past rear tergal margin; caudal margins of paraterga with five oblong projections (Fig. 7B). Lateral margins of paraterga beset with numerous, similarly oblong and usually subequal, setigerous tubercles/lobulations. Ozopores inconspicuous, opening dorsally near penultimate lateral lobulation on pore-baring rings 5, 7, 9, 10, 12, 13, 15–19. Strictures between pro- and metazona narrow and rather deep, nearly smooth.

Telson: Epiproct small, conical and subtruncate at tip. Paraprocts with 2+2 setae on triangular, projections/knobs (Figs 5C, 6C). Hypoproct trapeziform, with 1+1 setae borne on distinct oblong knobs at caudal margin.

Limbus very thin, small, and entire. Neither an axial line nor pleurosternal carinae (Fig. 7A–C). Endotergum inconspicuous, posterior margin of metazona projecting into long, sharp, apically microdenticulate, triangular projections (Fig. 7F).

Gonopodal aperture roundly pentagonal, relatively small, taking up ~ 1/2 width of metazonum 7, clearly open and drawn into metazonum 6.

Midbody legs incrassate, rather long. 1.4–1.5 × as long as body height, with small, stout, abundant and usually curved setae with admixture of sphaerotrichomes ventrally on all podomeres (♂, Fig. 7E); gonopores on ♂ coxae 2 inconspicuous, each borne on a very small swelling (♂); prefemora not bulged laterally; claws simple and very small; in length, tarsus > femur > prefemur > tibia > postfemur > coxa.

**Figure 8.**
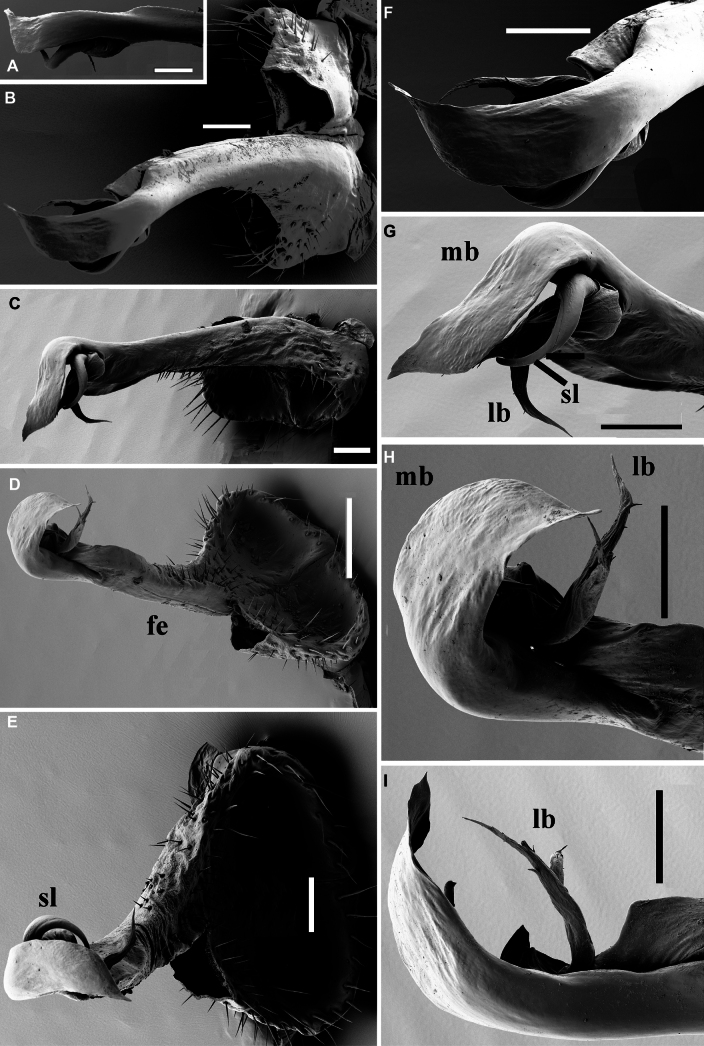
*Dalodesmushamatus* (Brandt, 1841), ♂ from Makira (ZFMK MYR13631). SEM micrographs. Gonopod, **A** = right **B–I** = left. **A** Right, anterior view **B** left, anterior view **C** lateral view **D** posteromesal view **E** posterior view **F** acropodite, anterior view **G** acropodite, lateral view **H** acropodite, posteromesal view **I** acropodite, mesal view. Abbreviations: fe = femorite; lb = lateral branch; mb = mesal branch; sl = solenomere branch. Scale bars: 100 µm.

Gonopods (Figs 6D–G, 8A–I) very slender and long, tips in situ reaching anteriorly until coxae 5. Both coxites and prefemorites (= densely setose parts of telopodites) equally very short and stout, fused medially, the former fully and the latter mostly hidden inside gonopodal aperture. Femorites (fe) contiguous medially in basal 1/3, setose almost all along, both slightly diverging distad towards acropodites. Apical portions of each telopodite (= acropodites) clearly diverging, rather simple and compact, curved ventrad and clearly divided into three unequal branches: a short, slightly curved, submesal, tubiform, simple and non-sigmoid solenomere (sl) flanked by a rather simple, 2-branched solenophore, this latter being represented by a flagelliform, slightly barbed, short and acuminate lateral branch (lb), and a particularly large, lobe-shaped, acuminate and membranous mesal branch (mb) with a tooth near base.

#### ﻿ Dalodesmus odontopezus

Taxon classificationAnimaliaPolydesmidaDalodesmidae

(Attems, 1898)

272301F9-3D9B-5E34-9317-C4940B710DBF

Fig. 9

Tubercularium odontopezum Attems, 1898: 360, plate 7, figs 158–161 (D).
Polydesmus (Tubercularium) odontopezum (sic!) – de Saussure and Zehntner 1902: 89 (D).
Tubercularium odontopezum – Attems 1940: 435, fig. 618 (D, K).
Dalodesmus odontopezum (sic!) – Hoffman 1974: 230 (L).
Dalodesmus odontopezus – Jeekel 1965: 238 (L); Golovatch and Hoffman 1989: 161 (L); Enghoff 2003: 623 (L); Wesener and Enghoff 2022: 926 (L).

##### Note.

**Figure 9.**
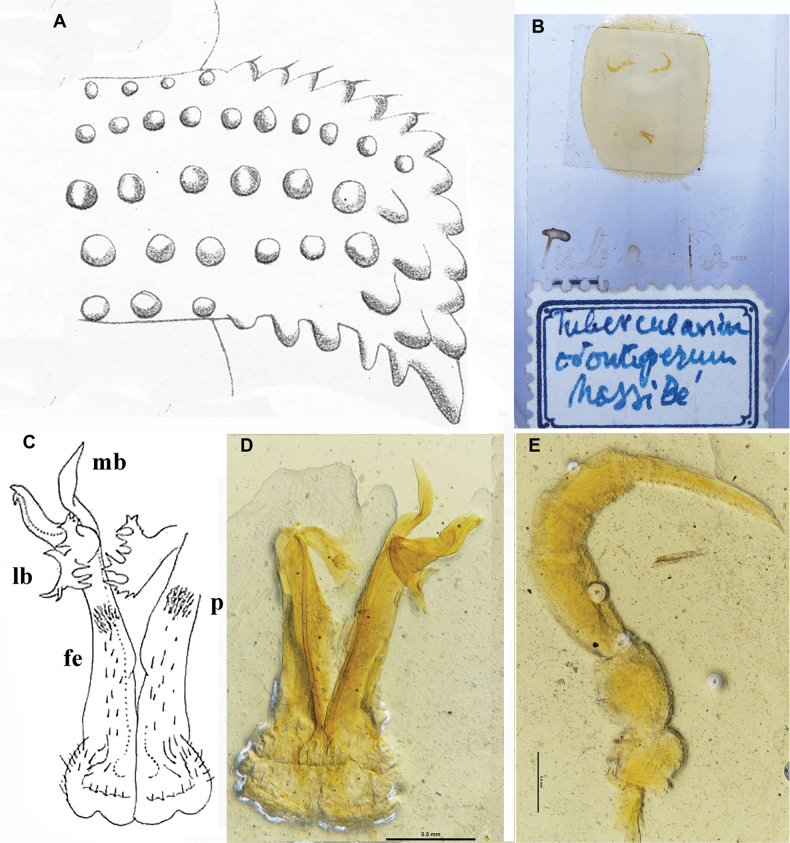
*Dalodesmusodontopezus* (Attems, 1898), ♂ holotype **A** right paratergum 14, dorsal view (after Attems 1898) **B** slide of holotype **C** both gonopods, ventral view (after Attems 1940) **D** both gonopods, ventral view (slide NHMW MY4429, photograph by O. Macek) **E** midbody leg. Abbreviations: lb = lateral branch; mb = mesal branch; pfe = prefemorite; sl = solenomere branch. Scale bars: 500 µm.

This species was described from a single ♂ holotype, from Nosy Be Islet (Attems 1898), slide NHMW MY4429 containing its two legs and gonopods (Fig. 9C), labelled *Tuberculariumodontopezum* (Attems, 1898), revised, in the NHMW collection. The torso seems to be misplaced.

##### Brief description.

(After Attems 1898, 1940.) ♂ holotype 28 mm long and 2.0 and 3.5 mm wide on pro- and metazona, respectively. Colouration of metaterga more intense brown in posterior halves, lighter in anterior ones; sides of paraterga yellowish, venter light yellowish brown, antennae and legs dirty yellow. Paraterga horizontal, not upturned above dorsum, sharpened caudally; dorsal tuberculations between paraterga mostly round (Fig. 9A).

Gonopods (Fig. 9B, C) showing setose femorites (fe), each with a small roundish, pulvillus-like field (p) of particularly dense setae ventrally near apex, coupled with complex acropodites: a slightly sigmoid and apically bifid solenomere (sl) lying between the highest, erect and subspiniform mesal branch (mb) and a much shorter, but prominent, roughly laciniate and fimbriate lateral branch (lb) directed ventrad.

#### ﻿ Dalodesmus orator

Taxon classificationAnimaliaPolydesmidaDalodesmidae

Hoffman, 1974

4F9FCBD8-F276-5C5B-B032-EDB13FA1CEE1

Fig. 10

Dalodesmus orator Hoffman, 1974: 225, figs 1–9 (D); Golovatch and Hoffman 1989: 162 (L); Enghoff 2003: 623 (L); Wesener and Enghoff 2022: 926 (L).

##### Remark.

This species was described from a ♂ holotype, two ♂ and two ♀ paratypes, all coming from Ambohimitombo, central Madagascar (Hoffman 1974), in the collection of the Natural History Museum in London, United Kingdom.

##### Brief description.

**Figure 10.**
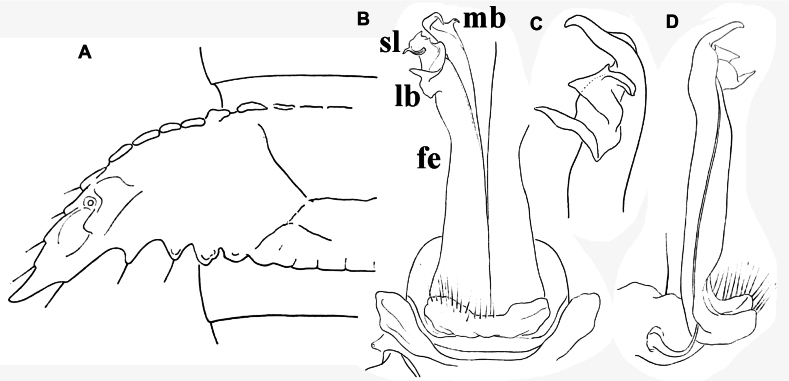
*Dalodesmusorator* Hoffman, 1974, ♂ holotype, modified from Hoffman 1974**A** midbody tergite, dorsal view **B** gonopod, posterior view **C** acropodite, mesal view **D** gonopod, lateral view. Abbreviations: lb = lateral branch; mb = mesal branch; pfe = postfemorite; sl = solenomere branch. Not to scale.

(After Hoffman 1974.) ♂ holotype ~ 26.5 mm long and up to 2.3 and 5.3 mm wide on pro- and metazona, respectively; ♀ up to 4.5 mm wide. Colouration light reddish brown with yellow paratergal apices and legs. Paraterga largely clearly upturned above dorsum and directed more laterad than caudad, at ca 45°, tips elongate and sharpened caudally; dorsal surface between paraterga mostly vaguely areate (Fig. 10A).

Gonopods (Fig. 10B–D) showing bare femorites (fe), coupled with rather complex acropodites: an unusually short and small solenomere (sl) directed ventrad, lying between the highest, suberect, distally unequally bifid mesal branch (mb) and a much shorter and strongly folded lateral branch (lb) directed ventrad.

#### ﻿ Dalodesmus tectus

Taxon classificationAnimaliaPolydesmidaDalodesmidae

Cook, 1896

6A41D93A-15FC-56E1-A607-D131B2351A9F

Figs 11 , 12

Dalodesmus tectus Cook, 1896: 26 (D); Attems 1940: 489 (L); Jeekel 1965: 238, figs 1, 2 (D); Hoffman 1974: 230 (D); Golovatch and Hoffman 1989: 161 (L); Enghoff 2003: 623 (L); Wesener and Enghoff 2022: 926 (L).
Polydesmus hova de Saussure & Zehntner, 1897: plate 5, figs 23–23c (D), syn. nov.
Pterodesmus hova – de Saussure and Zehntner 1901: 436 (D).
Polydesmus (Tubercularium) hova – de Saussure and Zehntner 1902: 91 (D).
Tubercularium hova – Attems 1940: 434, figs 619, 620 (D, K).
Dalodesmus hova – Jeekel 1965: 238 (L); Hoffman 1974: 230, figs 10, 11 (D); Golovatch and Hoffman 1989: 162, figs 7–9 (D); Enghoff 2003: 623 (L); Hollier and Wesener 2017: 58 (L, N); Wesener and Enghoff 2022: 926 (L).

##### Note.

**Figure 11.**
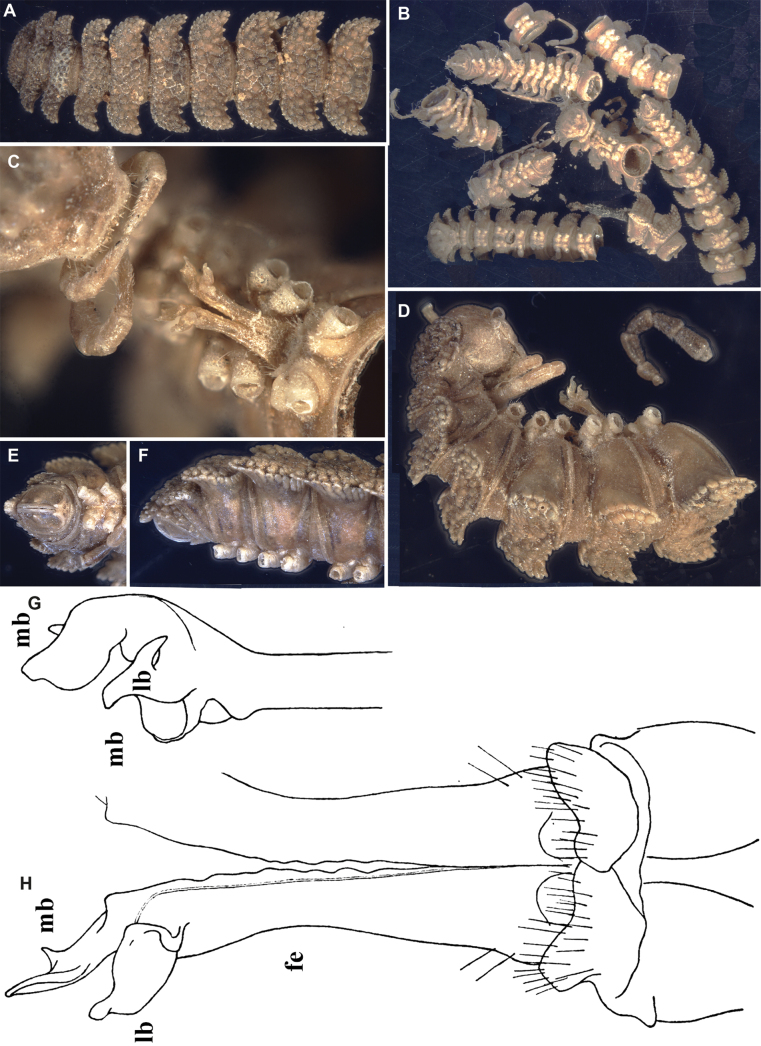
*Dalodesmustectus* Cook, 1896, ♂syntypes (ZMB MYR2110). Multi-layer photographs (**A–F**), drawings (after Hoffman 1974) (**G, H**). **A** Anterior body, dorsal view **B** both syntypes, fragmented mix **C** ventral view with gonopod **D** anterior body, lateral view with gonopod **E** telson, ventral view **F** telson, lateral view **G** gonopod, posterior view; gonopod, mesal view of acropodite. Abbreviations: fe = femorite; lb = lateral branch; mb = mesal branch. Not to scale.

This species was described from a ♂ holotype coming from an unspecified locality in central Madagascar (Cook 1896), in the ZMB collection, revised. The type series, ZMB MYR2110, actually contains two ♂ syntypes, one with dissected and missing gonopods, apparently the one depicted by Jeekel (1965), the second ♂ with still intact gonopods (Fig. 11A–F). Originally, *D.hova* was verbally described from an uncertain number of syntypes of both sexes (de Saussure and Zehntner 1901, 1902), with males from ‘Madagascar’ (coll. Sikora), as well as females and juveniles from Nosy Be Isle. Franz Sikora collected in Madagascar around the capital city Antananarivo and briefly in the southeast around Fort Dauphin. Hoffman (1974) recorded and illustrated the gonopods of *D.hova* from a ♂ taken as far away from part of the type locality (Nosy Be) as the Andasibe National Park (= Périnet) in eastern Madagascar, questioning such a vast and disjunct distribution. An incomplete ♂ syntype (with missing gonopods) from Nosy Be and 2 ♀ non-types from Nosy Sakatia Isle, all in the SMF collection, were later revised and partly depicted (Golovatch and Hoffman 1989). An additional six syntypes, collected by Franz Sikora, including the male on which the illustration of the gonopod was based in the original description, are in the MHNG collection (Hollier and Wesener 2017), as well as a non-type tube labeled “*Tuberculariumhova*”, “Madag. Fort Dauphin, S2 Remy 49; leg. Remy, det. Attems 1951”, in the NHMW collection.

Hoffman (1974) listed the type locality as Nosy Be, overlooking that the only male specimens used for the first description by de Saussure and Zehntner had come in fact from Franz Sikora (so either central Madagascar or Fort Dauphin in the southeast). We discovered another Malagasy Dalodesmidae species (see *Phymatodesmussakalava* above) collected by Franz Sikora that had actually come from Andasibe, exactly the same locality whence Hoffman briefly redescribed the species and finely illustrated its gonopodal structure. Thus, based on the female-based records of “*D.hova*”, *D.tectus* could be a congener particularly widely distributed across Madagascar, ranging from the isles of Nosy Be and Nosy Sakatia in the very north to Fort Dauphin (Tolagnaro) in the very southeast. If true, this seems to be the most widespread native millipede in Madagascar. The populations from Nosy Be and Fort Dauphin, from which the gonopods are currently unknown, should be carefully checked in the future to clarify their taxonomic status. Nosy Be is the type locality of another two Dalodesmidae, *D.odontopezus* and *Eutuberculariumvoeltzkowi*.

##### Brief description.

(After Cook 1896 and Jeekel 1965.) Body of ♂ syntypes ~ 21 mm long and 2.8 mm wide. Colouration uniformly dark brown. Paraterga largely subhorizontal, lying below dorsum; tips sharpened caudally, but not projecting past posterior margin; dorsal surface between paraterga mostly tuberculate, tuberculations being rounded to subconical (Fig. 11A).

Gonopods (Fig. 11C, D, G, H) showing nearly bare femorites (fe), both only basally with 2+2 lateral setae, coupled with rather simple acropodite: an untraceable, apparently rudimentary solenomere lying between both branches of a distinct solenophore: the highest, suberect, at midlength unequally bifid mesal branch (mb) and a much shorter, strongly folded and lamelliform lateral branch (lb).

**Figure 12.**
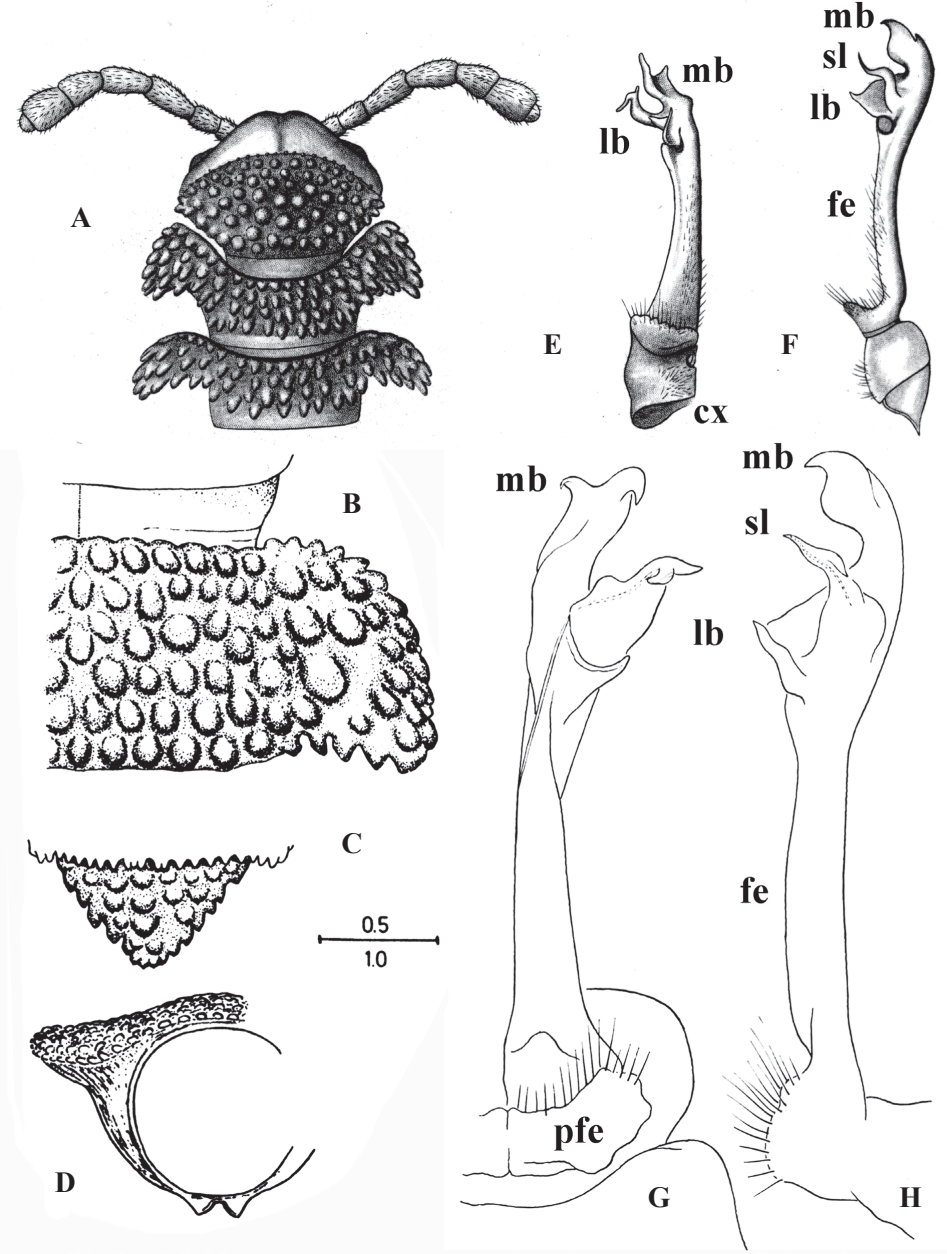
*Dalodesmustectus* Cook, 1896, syntypes of *D.hova* (de Saussure & Zehntner, 1897) syn. nov., ♀ syntype (**A**) and ♂ syntype (**E, F**) (after de Saussure and Zehntner 1902), ♂ syntype (**B–D**) (after Golovatch and Hoffman 1989), all from Nosy Be, and ♂ from Andasibe National Park (**E–H**) (after Hoffman 1974). **A** Anterior part of body, dorsal view **B** right half of metazonum 10, dorsal view **C** posterior end of body, dorsal view **D** body ring 9, oral view **E** right gonopod, ventral view **F** left gonopod, lateral view **G, H** left gonopod, ventral and lateral views, respectively. Abbreviations: cx = coxite, fe = femorite, lb = lateral branch, mb = mesal branch, sl = solenomere. Scale bar: 1000 µm (**B–D**).

Based on a restudy of the gonopods of the type series of *D.tectus*, no meaningful differences to the gonopodal structure of *D.hova* as illustrated by de Saussure and Zehntner (1897) and redescribed by Hoffman (1974) could be found (Fig. 12A–H). The potential type locality of *D.tectus* (central Madagascar) fits very well to the type locality of *D.hova* (Andasibe).

### ﻿Descriptions of new species

#### ﻿ Dalodesmus kompantsevi sp. nov.

Taxon classificationAnimaliaPolydesmidaDalodesmidae

B77BE40B-1CC8-5299-8AFA-F927C052F425

https://zoobank.org/B8043509-6990-4E62-99B8-CDFE90E3B656

Figs 13 , 14

##### Type material.

***Holotype*** • ♂ (ZMUM), Northern Madagascar, Antsiranana Prov., 4 km SW of Joffreville (= Ambohitra), Parc National Montagne d’Ambre, 12.51358°S, 49.183001°E, 900–1000 m a.s.l., tropical forest, 16–18.XII.2018, A. Kompantsev leg.

##### Other material.

• 1 ♀, (MZUF Fi-30A), Madagascar, Montagne d’Ambre, 900 m, c/o grande cascade, leg. 26 Sept. 1989, L. Bartolozzi & S. Taiti.

##### Diagnosis.

**Figure 13.**
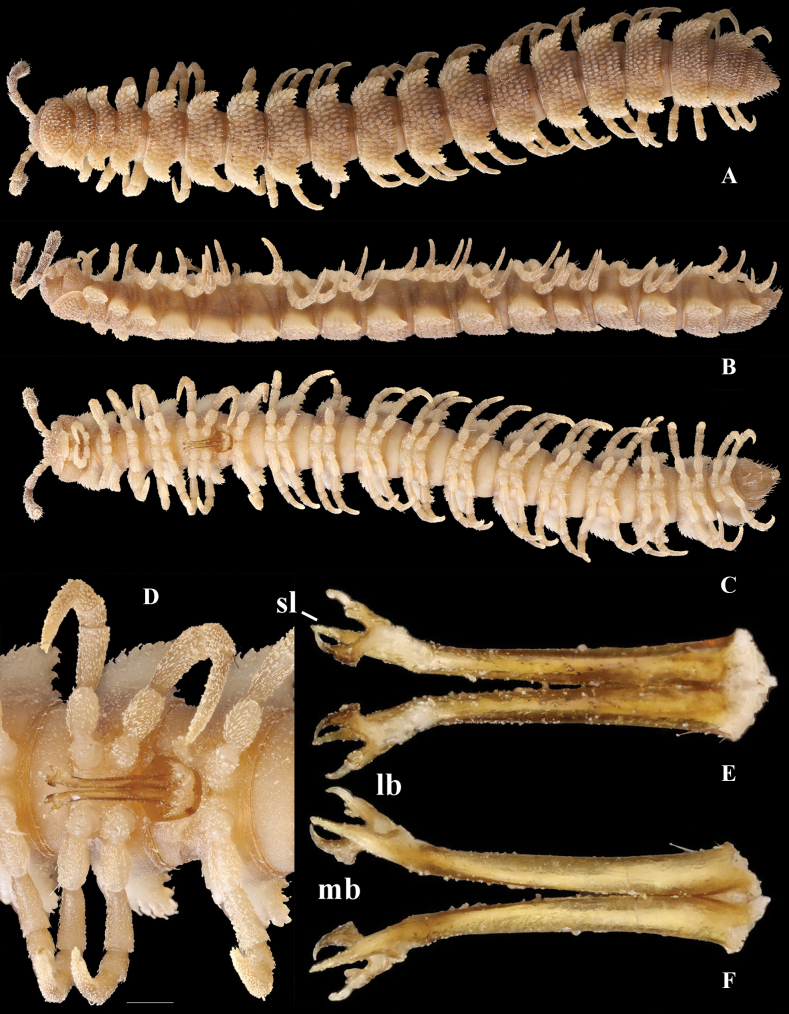
*Dalodesmuskompantsevi* sp. nov., ♂ holotype: **A–C** habitus, dorsal, lateral, and ventral views, respectively **D** body ring 7 with gonopods in situ, ventral view **E, F** gonopods, dorsal and ventral views, respectively. Abbreviations: fe femorite, lb lateral branch, mb mesal branch, sl solenomere. Photographs by K. Makarov, taken not to scale.

Tips of paraterga not projecting past posterior tergal margin (Fig. 13A–C), like in *D.hova*, *D.tectus* and *D.speophilus* sp. nov., vs sharper and projecting beyond margin in *D.hamatus*, *D.odontopezus* and *D.orator*. Differs from all other species of the genus primarily by the light colouration, coupled with the unusually compact, short, and clearly trifid acropodite, this being divided into three subequally long and upright branches: a middle, subacuminate and non-sigmoid solenomere (sl) flanked by a securiform, axe-shaped mesal branch (mb) and a nearly finger-shaped lateral branch (lb) of the solenophore (Fig. 13E, F). See also Key below.

##### Etymology.

To honour the late Aleksandr Kompantsev (Russia), the collector.

##### Description.

Length of holotype ~ 23 mm, width of midbody pro- and metazona 1.5 and 2.9 mm, respectively. Width of prozona 2.2 mm, of metazona 4.2 mm in ♀ non-type.

Colouration in alcohol uniformly pale brown to beige, axial stripe on prozona thin, vague, and grey, antennae somewhat infuscate, increasingly brown distad (Fig. 13A–C).

**Figure 14.**
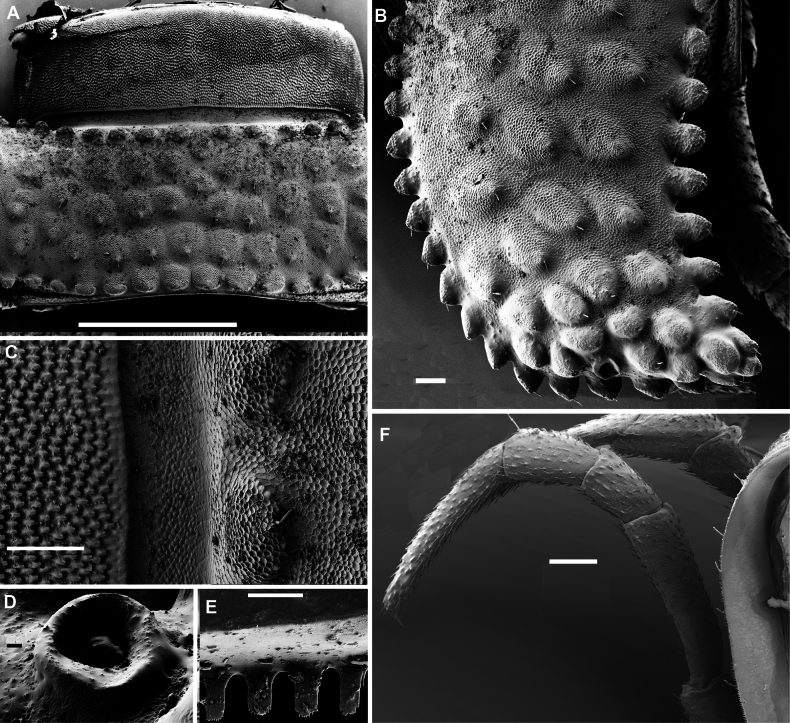
*Dalodesmuskompantsevi* sp. nov., ♀ (MZUF Fi30A). SEM micrographs **A** midbody ring with pro- and metazonite, dorsal view **B** midbody ring, paranotum with ozopore **C** detail of surface structures of prozonite and metazonite **D** ozopore **E** endotergum **F** midbody legs. Scale bars: 1000 µm (**A**); 100 µm (**B, C**); 10 µm (**D, E**); 200 µm (**F**).

Body with 20 rings. Tegument mainly dull to only slightly shining, microgranulate to microtuberculate throughout, even surfaces of prozona and of metazona below paraterga finely microgranulate, sterna granulate. Head also densely microtuberculate or granulate throughout, micropilose up to level of antennae; epicranial suture thin, but distinct; genae squarish, set off ventrally from gnathochilarial stipes by a small, but evident ridge (Fig. 13A–C). Interantennal isthmus ~ 2 × diameter of antennal socket (Fig. 13C). Antennae short and rather clavate, in situ reaching back past ring 3 when stretched dorsally, very densely setose and microgranulate. In length, antennomere 6 > 5 > 3 > 4 > 2 > 1 = 7; antennomere 6 the largest and the highest, antennomeres 5 and 6 each with a small, round, distodorsal knob. In width, collum < head < ring 3 < 2 = 4–16; thereafter body gradually tapering towards telson (Fig. 13A). Collum transversely suboval, regularly and broadly rounded laterally, densely tuberculate, most tuberculations being slightly oblong-oval, evident, equipped with very short, mostly subclavate setae and arranged in 7–8 transverse, rather irregular, arcuate rows. Metaterga 2 and 3 each with four similar transverse arcuated rows of setiferous tubercles, following metaterga each largely with 5–6 such rows (Figs 13A, 14A, B). Paraterga well-developed, set high (mostly at upper ¼ body), largely slightly upturned to subhorizontal, thus leaving the dorsum only faintly convex (Figs 13B, 14B); anterior and posterior margins of paraterga 2 and 3 clearly drawn both forward and caudad, following paraterga drawn increasingly only caudad, but caudal corners produced past rear tergal margin only on rings 17–19; posterior margins clearly bisinuate, well concave behind paraterga (Fig. 13A–C). Lateral and caudal margins of paraterga beset with numerous, similarly oblong, and usually subequal, setigerous tubercles/lobulations, caudolateral lobulation nearly sharp. Ozopores (Fig. 14B, D) inconspicuous, opening dorsally near penultimate lateral lobulation on pore-baring rings 5, 7, 9, 10, 12, 13, 15–19. Strictures between pro- and metazona narrow and rather deep, nearly smooth (Fig. 14C). Epiproct small, conical and subtruncate at tip. Hypoproct trapeziform, with 1+1 setae borne on distinct oblong knobs at caudal margin. Limbus very thin, small, and entire. Neither an axial line nor pleurosternal carinae (Fig. 13A–C). Endotergum inconspicuous, posterior margin of metazona projecting into long, sharp, apically microdenticulate, triangular projections (Fig. 14E).

Sterna mostly densely setose, deep, and narrow between coxae 1–3, increasingly broad thereafter, clearly excavate between coxae 5 and 6 (♂); postgonopodal sterna rather flat, devoid of modifications, cross-impressions faint (Fig. 13C). Gonopodal aperture roundly pentagonal, relatively small, taking up ~ 1/3 width of metazonum 7, clearly open and drawn into metazonum 6 (Fig. 13D). Legs incrassate, rather long. 1.4–1.5 × as long as body height, with small, stout, abundant and usual curved setae (Fig. 14F) with admixture of sphaerotrichomes ventrally on all podomeres (♂); gonopores on coxae 2 inconspicuous, each borne on a very small swelling (♂); prefemora not bulged laterally; claws very small; in length, tarsus > femur > prefemur > tibia > postfemur > coxa.

Gonopods (Fig. 13D–F) very slender and long, tips in situ reaching anteriorly beyond coxae 6 (Fig. 13C, D). Both coxites and clearly prefemoral (= densely setose) parts of telopodites equally very short and stout, fused medially, the former fully and the latter mostly hidden inside gonopodal aperture. Femorites (fe) contiguous medially in basal 1/3, basically bare and only one at base with a distinct lateral seta, both very faintly attenuating and diverging distad. Acropodites of both telopodites clearly diverging, but unusually compact, short, and clearly trifid, being divided into three subequally short and upright branches: a middle, subacuminate and non-sigmoid solenomere (sl) flanked by a solenophore represented by a securiform, axe-shaped mesal branch (mb) and a nearly finger-shaped lateral branch (lb) with a mesal knob parabasally.

#### ﻿ Dalodesmus speophilus sp. nov.

Taxon classificationAnimaliaPolydesmidaDalodesmidae

42B51489-FA57-5CB4-97D6-53CBD8BBF811

https://zoobank.org/F72BC885-741B-4E04-B34D-4C0DDD286655

**Figure 15.**
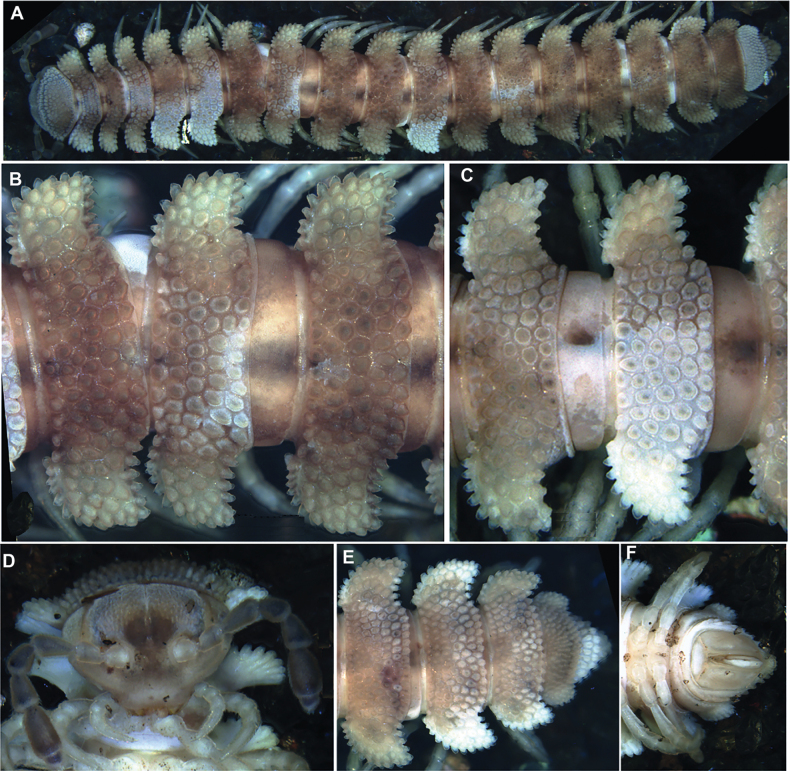
*Dalodesmusspeophilus* sp. nov., ♂ holotype (MZUF), multi-layer photographs **A** habitus, dorsal view **B** midbody rings, dorsal view **C** posterior body rings, dorsal view **D** head, ventral view **E** posterior body rings with telson, dorsal view **F** telson, ventral view. Not to scale.

**Figure 16.**
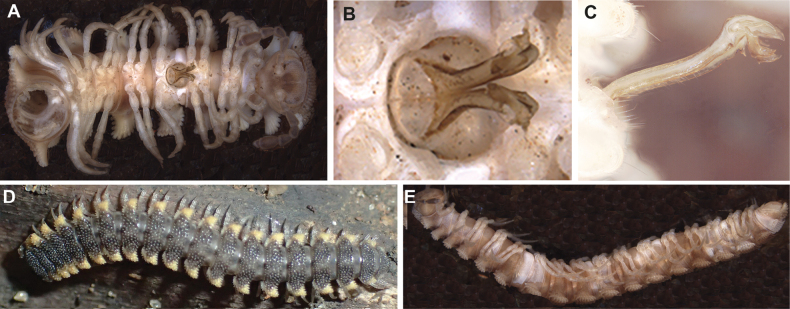
*Dalodesmusspeophilus* sp. nov., **A–C, E** ♂ holotype (MZUF), multi-layer photographs **D** photograph of live specimen of Dalodesmuscf.speophilus, courtesy Justin Gerlach. **A** Anterior part of body with gonopods, ventral view **B** gonopods, posterior view **C** gonopods, lateral view **D** live specimen **E** habitus, lateral view. Not to scale.

**Figure 17.**
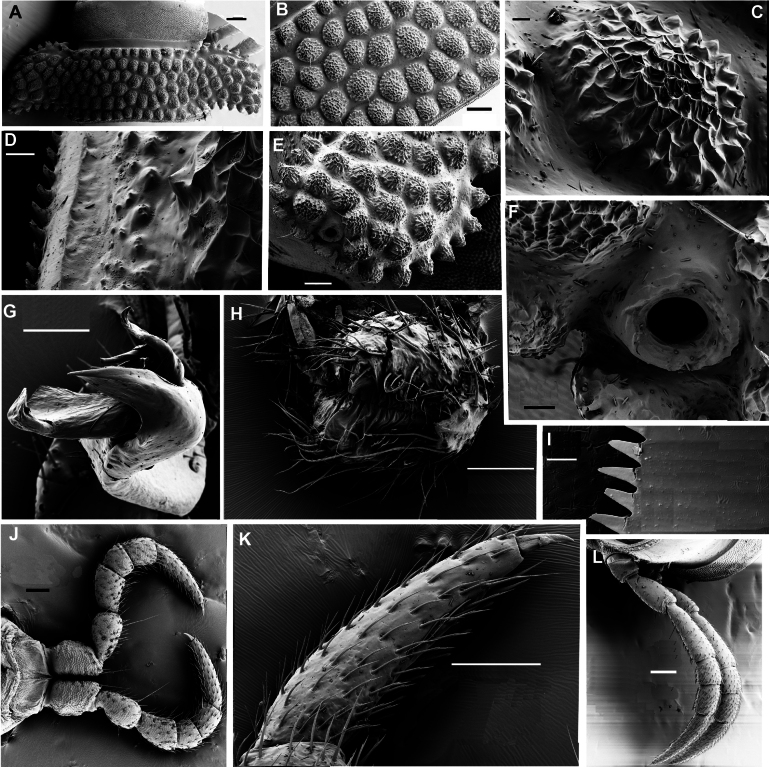
*Dalodesmusspeophilus* sp. nov., ♂ holotype (MZUF), ♀ paratype (ZFMK), SEM micrographs **A** midbody ring with pro- and metazonite, dorsal view **B** surface of metazonite **C** detail of surface structures of metazonite **D** endotergum **E** paratergum with ozopore **F** ozopore **G** gonopod, apical view **H** female vulva; **I** endotergum, ventral view **J** male leg pair 1, posterior view **K** tarsus 1 **L** midbody legs. Scale bars: 200 mm (**A, L**); 100 µm (**B, E, G, H, J, K**); 10 µm (**C; D, I**); 20 µm (**F**).

**Figure 18.**
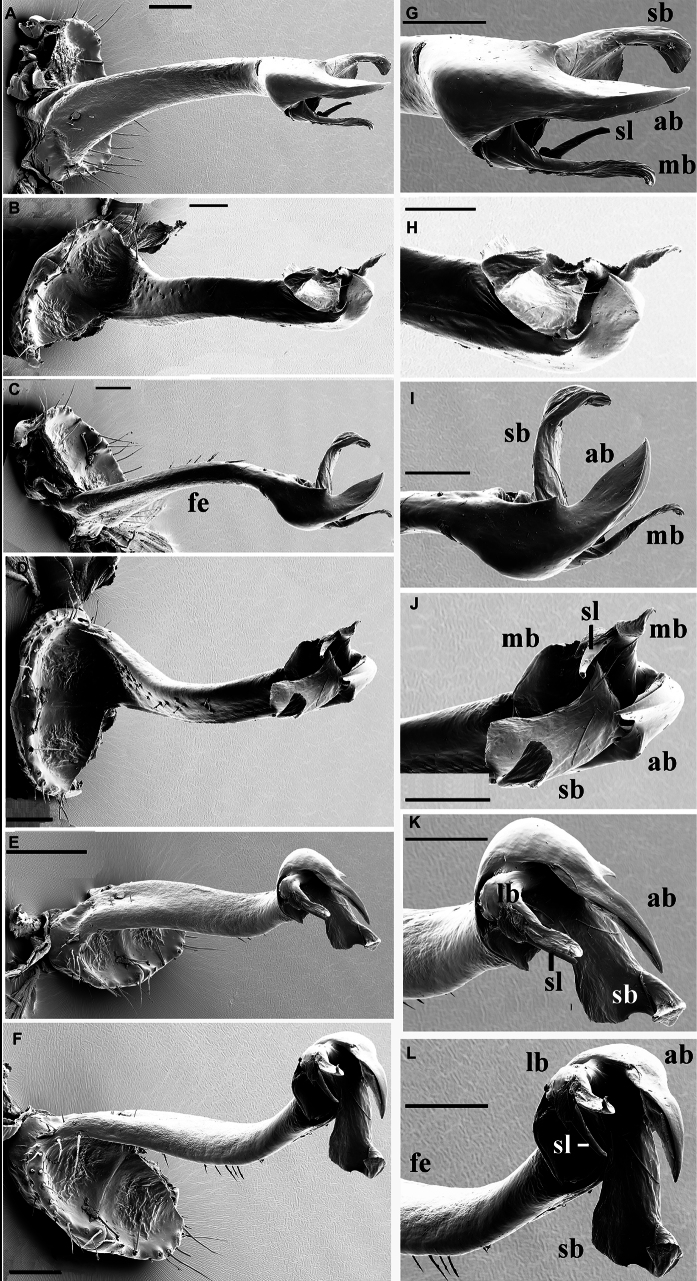
*Dalodesmusspeophilus* sp. nov., ♂ holotype (MZUF), SEM micrographs, right gonopod **A** anterior view **B** postero-mesal view **C** mesal view **D** posterior view **E** lateral view **F** lateral view **G** acropodite, anterior view **H** acropodite, postero-mesal view **I** acropodite, mesal view **J** acropodite, posterior view **K** acropodite, lateral view **L** acropodite, lateral view. Abbreviations: ab = apical branch; fe = femorite; lb = lateral branch; mb = mesal branch; sb = subapical branch; sl = solenomere branch. Scale bars: 100 µm.

Figs 15 , 16 , 17 , 18

##### Type material.

***Holotype*** • ♂ (MZUF), Madagascar, Grotta di Anjohibe, 15°32'33.08"S, 46°53'5.99"E, 12.ix.1989, L. Bartolozzi & S. Taiti leg.; ***Paratypes***: 6 ♂, 12 ♀, 12 juv. (MZUF); 1 M, 1 F (ZFMK), same data as holotype.

##### Diagnosis.

Length < 20 mm in both sexes, the smallest species of the genus (all other species with males > 21 mm). Tips of paraterga not projecting past posterior tergal margin, like in *D.hova*, *D.tectus*, *D.kompantsevi* sp. nov., vs sharper and projecting beyond margin in *D.hamatus*, *D.odontopezus* and *D.orator*. Differs from *D.hova*, *D.tectus*, and *D.kompantsevi* sp. nov. in the contrasting yellow paratergal tips (uniformly brown in the other species). Gonopods unique for the genus *Dalodesmus* in the main branch being subdivided into an apical and a subapical branch. See also Key below.

##### Etymology.

From *speophilus*, a noun in apposition, after the type locality, a gigantic cave.

##### Description.

Length in male ~ 16.5–17.5 mm (*n* = 3), width of midbody pro- and metazona 1.5 and 2.8–2.9 mm (*n* = 3), respectively, female length 16.5–19 mm (*n* = 4), width of prozona 1.6 mm (*n* = 1), of metazona 3.7–4.1 mm (*n* = 4).

Colouration in alcohol light brown to beige, paraterga light, head brown, epicranium grey, legs light, except for a few tarsi pale grey; antennae dark brown (Fig. 15A–F). A live photograph of a potential *D.speophilus* sp. nov., 70 km away from the type locality, shows dull, dark brown to blackish tergites with pale white to almost yellow paratergal tips, legs white to pale grey (Fig. 16D).

Body with 20 rings. Tegument mainly dull, microgranulate to microtuberculate throughout, even surfaces of prozona and of metazona below paraterga finely microgranulate, sterna granulate. Head also densely microtuberculate or granulate throughout, micropilose up to level of antennae; epicranial suture thin, but distinct; genae squarish, set off ventrally from gnathochilarial stipes by a small, but evident ridge (Figs 15D, 16A). Interantennal isthmus ~ 2 × diameter of antennal socket (Fig. 15D). Antennae short and rather clavate, in situ reaching in both sexes back past ring 3 when stretched dorsally, very densely setose and microgranulate. In length, antennomere 6 > 2 > 5 > 4 > 2 > 1 = 7; antennomere 6 the largest and the highest, antennomeres 5 and 6 each with a small, round, distodorsal knob, most likely beset with sensory cones. In width, collum ≤ head < ring 3 < 2 < 4–16; thereafter body gradually tapering towards telson (Fig. 15A). Collum transversely suboval, regularly and broadly rounded laterally, densely tuberculate, most tuberculations being slightly oblong-oval, evident, equipped with very short, mostly subclavate setae and arranged in 20–22 lateral, 7–8 transverse, rather irregular, arcuated rows. Metaterga 2 and 3 narrow, each with four similar, transverse, arcuated rows of setigerous tubercles (Fig. 17C), following metaterga each largely with 5–6 such rows (Fig. 15A). Paraterga well-developed, set high (mostly at upper ¼ body), largely slightly upturned to subhorizontal, thus leaving the dorsum only faintly convex (Fig. 15A–C); anterior and posterior margins of paraterga 2 and 3 clearly drawn both forward and caudad, following paraterga drawn increasingly only caudad, but caudal corners produced past rear tergal margin only on rings 15–19; posterior margins clearly bisinuate, well concave behind paraterga (Fig. 15E). Lateral and caudal margins of paraterga beset with numerous, similarly oblong and usually subequal, setigerous tubercles/lobulations, caudolateral lobulation being nearly sharp (Fig. 17A, B). Ozopores inconspicuous (Fig. 17E, F), opening dorsally near penultimate lateral lobulation on pore-baring rings 5, 7, 9, 10, 12, 13, 15–19. Strictures between pro- and metazona narrow and rather deep, nearly smooth (Fig. 17A).

Telson: Epiproct small, conical and subtruncate at tip. Hypoproct trapeziform, with 1+1 setae borne on distinct oblong knobs at caudal margin. Paraprocts with 2+2 setae on triangular, projecting knobs (Figs 15E, F, 16E).

Limbus very thin, small, and entire. Neither an axial line nor pleurosternal carinae (Fig. 15A–C). Endotergum inconspicuous, posterior margin of metazona projecting into dense, long sharp, apically microdenticulate, triangular projections (Fig. 15D, I).

Gonopodal aperture roundly pentagonal, relatively small, taking up ~ 1/2 width of metazonum 7, clearly open and drawn into metazonum 6 (Fig. 16A).

First ♂ leg-pair shorter and wider than other legs, with long coxae, large; size of its podomeres: tarsus > coxa > prefemur = femur > postfemur = tibia (Fig. 17J), tarsus with sharp claw, ventral spines, and numerous long setae (Fig. 17K). Midbody legs incrassate, rather long. 1.4–1.5 × as long as body height (Fig. 17L), with small, stout, abundant, and usually curved setae with admixture of sphaerotrichomes ventrally on all podomeres (♂); gonopores on ♂ coxae 2 inconspicuous, each borne on a very small swelling (♂); prefemora not bulged laterally; claws very small; in length, tarsus > femur > prefemur > tibia > postfemur > coxa.

♀ vulva setose, symmetrical, lateral and inner plates of same width. Operculum large, each side with three or four very long setae, longest reaching the apical margin of vulva (Fig. 17H).

Gonopods (Figs 16B, C, 17G, 18A–L) very slender and long, tips in situ reaching anteriorly until coxae 5 (Fig. 16A, B). Both coxites and prefemoral (= densely setose parts) of telopodites equally very short and stout, fused medially, the former fully and the latter mostly hidden inside gonopodal aperture. Femorites (fe) contiguous medially in basal 1/3, sparsely setose almost all along, both slightly flattened dorsoventrally and diverging distad towards acropodites. Apical portions of telopodite (= acropodites) clearly diverging, rather complex and compact, curved ventrad and clearly divided into four unequal branches: a short, submesal, tubiform, simple and non-sigmoid solenomere (sl) flanked by a rather elaborate, tri-branched solenophore, this latter being represented by an immediately adjacent, short, lobe-shaped, lateral branch (lb), a large, subacuminate, slender, twisted, and membranous medial branch (mb) with a denticle near base, and an apical (ab) and a subapical branch (sb), both latter membranous, curved/bent ventrad and ragged/irregular in shape.

### ﻿Unidentified Dalodesmidae from Madagascar

In order to provide a better overview of the distribution, permitting future collection efforts to be more successful, all unidentified but available material of the family Dalodesmidae is listed below. The morphological description of these samples is beyond the scope of this study.

#### ﻿*Dalodesmus* spp.

1 F, **CAS DSD006**, Madagascar, Toamasina, Prov., Parc National Masoala, Ambohitsitondroina Mt., Ambanizana, rainforest, 650 m, 15°34'10"S, 050°00'12"E, coll. 26 Feb – 06 Mar 2003, Andriamalala, Silva et al. general collecting day, at campsite; 1 F, **CAS DSD0016**, same data as previous, but raking tree trunks; 1 M, CAS DSD0023, Madagascar, Toamasina, Prov., Parc National Masoala, Ambohitsitondroina Mt., Ambanizana, rainforest, 900–9502 m, 15°34'20"S, 050°00'25"E, coll. 5 March, 2003, Andriamalala, Silva et al., sweeping low herbs; 1 M, **CAS DSD0027**, Madagascar, Toamasina Prov., Parc National Masoala, Ambohitsitondroina Mt., Ambanizana, montane rainforest, 1010 m, 15°34'27"S, 050°00'39"E, coll. 6 March, 2003, Andriamalala, Silva et al., beating low vegetation; 2 F, **CAS DSD0037**, Madagascar, Antananarivo, Prov., R. S. d’Ambohitantely, Forêt d’Ambohitantely, ca. 20.9 km 72° NE, Ankazobe, primary forest, 1574 m, 18°13'30"S, 047°16'44"E, coll. 19 March 2003, Andriamalala, Silva et al., raking tree trunks; 3 ?, **CAS DSD0045**, Madagascar, Antananarivo, Prov., R. S. d’Ambohitantely, Forêt d’Ambohitantely, 20 km NE Ankazobe, forest fragment, montane rainforest, 1638 m, 18°12'30"S, 047°17'08"E, coll. 20 March 2003, Andriamalala, Silva et al., raking tree trunks; 1 M, 1 F, 1 juvenile, **CAS BLF7917**, Madagascar, Toliara Province, Fôret Classée d’Analavelona, 33.2 km 344° NNW Mahaboboka, montane rainforest, 1300 m, 22°38'34"S, 044°10'16"E, coll 22–26 Feb 2003, Fisher, Griswold et al., beating low vegetation; 8 M & F, **CAS BLF8151**, Toamasina, Montagne d’Anjanaharibe, 19.5 km 27° NNE Ambinanitelo, montane rainforest, 1100 m, 15°10'42"S, 049°38'06"E, coll. 12–16 Mar 2003, Fisher, Griswold et al., beating low vegetation; 1 M, **CAS BLF9558**, Madagascar, Antsiranana, Forêt de Binara, 7.5 km 230°, SW Daraina, tropical semi-dry forest, 375 m, 13°15'8"S, 049°37'00"E, coll. 1 December 2003, B.L. Fisher, pitfall trap; 1 F, **MZUF Fi24, Mag1107**, Madagascar, RNI Andohahela, particella 1, versante E, forest pluviale, lettiera vagliata, 300 m, [24°57'S, 46°43'N] coll. 24.–26.v.1991, L. Bartolozzi, S. Taiti, C. Raharimina; 1 immature specimen, “Mg5” **NHMW**, Madagascar, “Forststation Manjakatompo bei Ambatolampy, Ankaratra- Massiv, [19°22'S, 47°18'E] Gesiebe aus morschem Holz und Strauch am Fuße großer Bäume u . a. Weinmannia spec., 12.04.1969”, Franz leg; 1 immature specimen, “Mg42” **NHMW**, Madagascar, “Galeriewald von Berenty [25°00'S, 46°18'E], Gesiebe aus morschem Holz und Fomes, Gesiebe aus schimmelnder Laubstreu, 02.05.1969”, Franz leg.; 1immature specimen, “Mg43” **NHMW**, Madagascar, “Straße nach Ft. Dauphin, 44 km von dort, [25°00'5.85"S, 46°36'28"E] Gesiebe aus Laubstreu neben Bach und Bachdetritus, 02.05.1969”, Franz leg.; 2 immature specimens, “Mg62” **NHMW**, “Madagascar, Montagne d‘Ambre, unterhalb der Forststation, [12°30'S, 49°11'E], Gesiebe unter faulenden Baumstämmen, und aus Laubstreu, 20.05.1969”, Franz leg.

#### ﻿Dalodesmidae, unidentified genus (*Phymatodesmus*?):

1 F, **ZFMK MYR13919**, Madagascar, Toliara Prov., Ambatotsirongorongo Mountain, Grande Lavasoa, rainforest, 500 m, 25°5'10.23"S, 46°44'55.93"E, coll. 14.vi.2007, T. Wesener & K. Schütte.

### ﻿Key to Dalodesmidae species of Madagascar

**Table:** 

1	Body length usually > 20 (17–28) mm. Paraterga well-developed, arcuated, apically pointed (Fig. 6B). Metatergal surface with often irregular, large, oval, piligerous tuberculations (Fig. 7A–D). Antennae long, protruding back to body ring 3 (Fig. 13A). Paraprocts with 2+2 setae located directly on their surface	**2**
–	Body length ~ 10 mm. Paraterga short, rectangular (Fig. 1A, D, H). Metatergal surface with regular, circular piligerous tuberculations (Figs 1H, 2A–E), at low magnification appearing to be pointed posteriorly due to the setae (Fig. 1G). Antennae short, protruding back to beginning of ring 2 (Fig. 1C). Paraprocts with 2+2 setae located on knobs	**Genus *Phymatodesmus*, one species: *P.sakalava* (Central, Eastern Madagascar: Andasibe)**
2	Gonopodal femorites long and slender, > 2 × as long as acropodites (Fig. 5D–G). Femorites diverging in distal half (Fig. 5D). Femorites bare or poorly setose ventrally and laterally (Fig. 5D–G)	**Genus *Dalodesmus* (6 species) (3)**
–	Gonopodal femorites stout, twice as long as acropodites (Fig. 4B). Femorites parallel dorsoventrally (Fig. 4B). Femorites densely setose both ventrally and laterally (Fig. 4B, C)	**Genus *Eutubercularium* , single species: *E.voeltzkowi* (Nosy Be)**
3	Paratergal tips rather sharp and narrow, projecting past posterior tergal margin (Fig. 5B)	**4**
–	Paratergal tips blunt and wide, not projecting past posterior tergal margin (Fig. 11A)	**6**
4	Body dark, paratergal tips contrasting yellow. Males > 25 mm	**5**
–	Body uniformly brown or black (Fig. 6A–C), paratergal tips of same colour. Males 20–23 mm long. Tegument either as usual, strongly calcified, and body rigid, or poorly calcified, and body unusually fragile. Gonopodal femorites setose (Fig. 6D–F), main branch broad apically, single-tipped; lateral branch bifid, narrow (Fig. 8A–I)	***Dalodesmushamatus* (‘Madagascar’, Makira).**
5	Paraterga not upturned. Gonopodal femorites setose (Fig. 9B), solenomere branch (sl) lying between single-tipped mesal branch (mb) and lateral branch (lb), lateral branch laciniate and fimbriate (Fig. 9B)	***Dalodesmusodontopezus* (Nosy Be).**
–	Paraterga upturned. Gonopod femorites bare (Fig. 10B), solenomere branch (sl) lying between bifid mesal branch (mb) and lateral branch (lb), lateral branch strongly folded (Fig. 10C, D)	***Dalodesmusorator* (Ambohimitombo).**
6	Paratergal tips same colour as terga, pale to chocolate brown (Fig. 13A). Both sexes > 20 mm long (males 21–23 mm). Gonopod without a subdivided medial branch	**7**
–	Paratergal tips contrasting yellow (Fig. 16D). Both sexes < 20 mm long (16.5–19 mm). Gonopod with a medial branch (mb) carrying a basal denticle and subdivided into an apical (ab) and a subapical (sb) branch (Fig. 18A–L)	***Dalodesmusspeophilus* sp. nov. (NE Madagascar)**
7	Dark brown or chocolate brown (Fig. 11A). Solenomere branch (sl) very short (Fig. 11G, H) or even rudimentary (Fig. 12G, H). Medial branch bifid, lateral branch folded	***Dalodesmustectus* (Andasibe, “Central Madagascar”, Nosy Be?, Fort Dauphin?)**
–	Pale brown (Fig. 13A). Solenomere branch (sl) as long as lateral branch (lb) and medial branch (mb). Gonopod uniquely trifid (Fig. 13E, F)	***Dalodesmuskompantsevi* sp. nov. (Montagne d’Ambre).**

## ﻿Conclusions

Dalodesmidae seem to be rarely encountered on Madagascar. There are very few photographic posts on iNaturalist of the group, especially compared to other large millipedes. A leaf litter sifting project (Spelzhausen et al. 2020) only captured one specimen, and in fact most of the collection samples examined by us only have few individuals (1 or 2, rarely 3), with the sole exception of the Grotte d’Anjohibe (*D.speophilus* sp. nov.), which is a very special habitat (a cave in a dry forest during the dry season). On a positive note, Malagasy Dalodesmidae millipedes seem to be capable of survival in old secondary forests next to natural woodlands (the *Eucalyptus* plantation planted in 1909 with many indigenous species in Andasibe, as well as the Manjakatompo forest). From the hundreds of Madagascar samples at the CAS and Field Museum (pitfall traps and Winkler extractions for ants), we only have nine collection events of Dalodesmidae.

**Figure 19.**
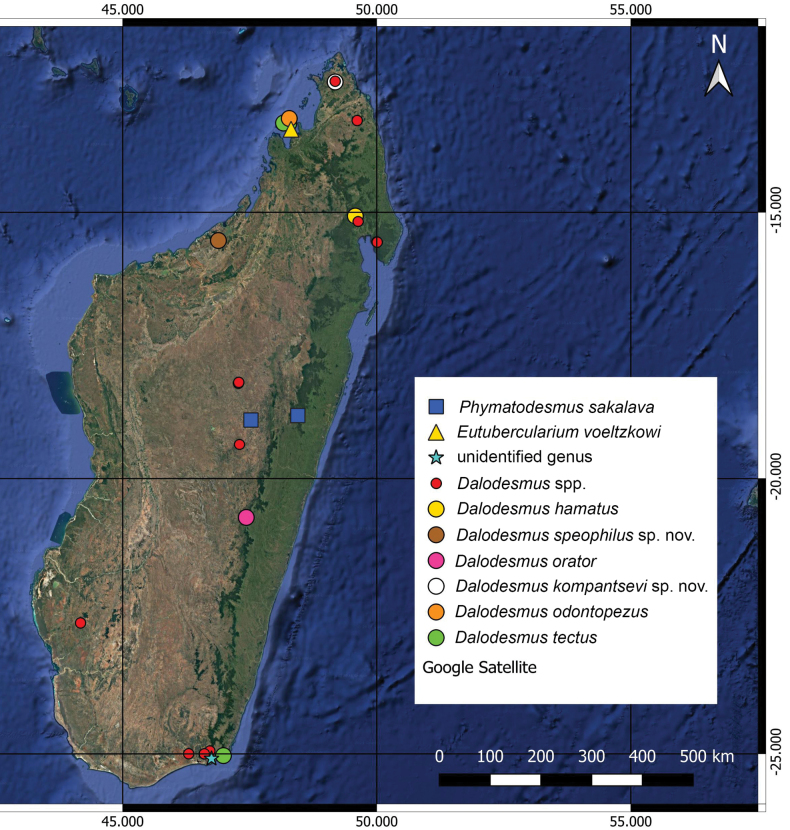
Distribution of Dalodesmidae in Madagascar.

All encounters of Dalodesmidae on Madagascar, including unidentified samples, mapped in Fig. 19, show that the family occurs virtually all over the island except for dry spiny forests. Yet it seems especially characteristic of woodlands, this being true of most Diplopoda of Madagascar and elsewhere. In millipedes, both the generic and, especially, the specific diversity seems to be higher in the northern part of Madagascar, gradually being reduced to the south. As a result of the absence of adult male material, numerous samples still remain unidentified to species, or even genus-level. Further taxonomic research on Malagasy Dalodesmidae may well reveal not only new records, but even new species. In addition, molecular studies would be very helpful to refine the picture in terms of both fauna and distribution.

## Supplementary Material

XML Treatment for Phymatodesmus

XML Treatment for Phymatodesmus sakalava

XML Treatment for Eutubercularium

XML Treatment for Eutubercularium voeltzkowi

XML Treatment for Dalodesmus

XML Treatment for Dalodesmus hamatus

XML Treatment for Dalodesmus odontopezus

XML Treatment for Dalodesmus orator

XML Treatment for Dalodesmus tectus

XML Treatment for Dalodesmus kompantsevi

XML Treatment for Dalodesmus speophilus

## ﻿Additional information

### Author ORCIDs

Thomas Wesener https://orcid.org/0000-0002-2028-3541

Nesrine Akkari https://orcid.org/0000-0001-5019-4833

### Data availability

All of the data that support the findings of this study are available in the main text.

## References

[B1] Attems C (1898) System der Polydesmiden. I. Theil. Denkschriften der Kaiserlichen Akademie der Wissenschaften zu Wien, Mathematisch-Naturwissenschaftliche Classe 67: 221–482.

[B2] Attems C (1940) Myriapoda 3. Polydesmoidea III. Fam. Polydesmidae, Vanhoeffeniidae, Cryptodesmidae, Oniscodesmidae, Sphaerotrichopidae, Peridontodesmidae, Rhachidesmidae, Macellolophidae, Pandirodesmidae. Das Tierreich 70, i– xvii+1–577. 10.1515/9783111609645.1

[B3] Brandt JF (1841a) Note supplémentaire sur les espèces qui composent le genre *Polydesmus*, suivie d’une caractéristique de deux espèces nouvelles. Bulletin scientifique publié par l’Académie Impériale des Sciences de St.-Pétersbourg 9(1): 9–11.

[B4] Brandt JF (1841b) Recueil de mémoires relatifs à l’ordre des Insectes Myriapodes et lus à l’Académie Impériale des Sciences de St.-Pétersbourg. Leipsik, 189 pp.

[B5] Brölemann HW (1916) Essai de classification des polydesmiens (Myriapodes). Annales de la Société Entomologique de France 84(4): 523–617. 10.1080/21686351.1915.12279415

[B6] Cook OF (1896) *Cryptodesmus* and its allies. Brandtia 5: 19–28.

[B7] de Saussure H Zehntner L (1897) Atlas de l’histoire naturelle des Myriapodes. In: Grandidier A (Ed.) Histoire physique, naturelle et politique de Madagascar. Paris: Imprimérie nationale, Pl 1–15.

[B8] de Saussure H Zehntner L (1901) Myriopoden aus Madagaskar und Zanzibar, gesammelt von Dr. A. Voeltzkow. Abhandlungen der Senckenbergischen naturforschenden Gesellschaft 26(4): 429–460.

[B9] de Saussure H Zehntner L (1902) Myriapodes de Madagascar. In: Grandidier A (Ed.) Histoire physique, naturelle et politique de Madagascar.Imprimérie nationale, Paris, 1–356.

[B10] Enghoff H (2003) Diplopoda, millipedes. In: Goodman SM Benstead JP (Eds) The Natural History of Madagascar.University of Chicago Press, Chicago and London, 617–627.

[B11] Enghoff H Golovatch SI Short M Stoev PE Wesener T (2015) Diplopoda – taxonomic overview. In: Minelli A (Ed.) Treatise on Zoology – Anatomy, Taxonomy, Biology.The Myriapoda. Volume 2. Brill, Leiden, 363–453. 10.1163/9789004188273_017

[B12] Golovatch SI Hoffman RL (1989) Identity of *Polydesmushamatus* Brandt 1841, a Malagasy milliped (DiplopodaPolydesmidaDalodesmidae). Tropical Zoology 2: 159–164. 10.1080/03946975.1989.10539436

[B13] Golovatch SI Hoffman RL (2000) On the diplopod taxa and type material of J. F. Brandt, with some new descriptions and identities (Diplopoda). Fragmenta Faunistica 43(Supplement): 229–249.

[B14] Hoffman RL (1974) Short studies on dalodesmid millipeds from South Africa and Madagascar. Wasmann Journal of Biology 32(2): 221–246.

[B15] Hoffman RL (1980) Classification of the Diplopoda. Muséum d’histoire naturelle, Geneva, 237 pp.

[B16] Hollier J Wesener T (2017) The Diplopoda (Myriapoda) of Madagascar described by Henri de Saussure and Leo Zehntner. Revue suisse de Zoologie 124(1): 53–65. 10.5281/zenodo.322665

[B17] Jeekel CAW (1965) The identity of *Dalodesmustectus* Cook, 1896, and the status of the family names Dalodesmidae Cook, 1896, Vanhoeffeniidae Attems, 1914 and Sphaeriotrichopodidae Attems, 1914 (Diplopoda, Polydesmida). Entomologische Berichten 25: 236–239.

[B18] Jeekel CAW (1971) Nomenclator generum et familiarum Diplopodorum: A list of the family and genus-group names in the Class Diplopoda from the 10^th^ edition of Linnaeus, 1758, to the end of 1957. Monografieën van de Nederlandse Entomologische Vereniging 5: 1–412.

[B19] Mesibov R (2017) Annotated catalogue of South American Dalodesmidae (Diplopoda, Polydesmida). Zootaxa 4338(3): 507–525. 10.11646/zootaxa.4338.3.6 29245715

[B20] Mesibov R Wesener T Hollier J (2018) A replacement name for *Dalodesmussakalava* (de Saussure & Zehntner, 1901) (Diplopoda: Polydesmida: Dalodesmidae). Zootaxa 4413(2): 389–391. 10.11646/zootaxa.4413.2.11 29690117

[B21] Spelzhausen L Wesener T Schütte K (2020) Vegetation thresholds for the occurrence of millipedes (Diplopoda) in different tropical forest types in Andasibe, Madagascar. Madagascar Conservation and Development 15(1): 1–8. 10.4314/mcd.v15i1.3

[B22] Wesener T Enghoff H (2022) Diplopoda, millipedes, *Menavetraka*, *Ankodabitra*, *Ankodiavitry*, *Marotanana*, *Sakolavitsy*. In: Fisher BL Goodman SM (Eds) The New Natural History of Madagascar.Princeton University Press USA, 918–933. 10.2307/j.ctv2ks6tbb.118

